# A necroptotic-independent function of MLKL in regulating endothelial cell adhesion molecule expression

**DOI:** 10.1038/s41419-020-2483-3

**Published:** 2020-04-24

**Authors:** Jialin Dai, Chonghe Zhang, Lin Guo, Hao He, Kai Jiang, Yingying Huang, Xixi Zhang, Haibing Zhang, Wu Wei, Yaoyang Zhang, Lihua Lu, Junhao Hu

**Affiliations:** 1 Interdisciplinary Research Center on Biology and Chemistry, Shanghai Institute of Organic Chemistry, Chinese Academy of Sciences, Shanghai, China; 2 University of Chinese Academy of Sciences, Beijing, China; 3 CAS Key Laboratory of Computational Biology, CAS-MPG Partner Institute for Computational Biology, Shanghai Institute of Nutrition and Health, Shanghai Institutes for Biological Sciences, University of Chinese Academy of Sciences, Chinese Academy of Sciences, Shanghai, China; 4 CAS Key Laboratory of Nutrition, Metabolism and Food Safety, Shanghai Institute of Nutrition and Health, Chinese Academy of Sciences, Shanghai, China; 5 Department of Neonatology, Shanghai First Maternity and Infant Hospital, Tongji University School of Medicine, Shanghai, China

**Keywords:** Necroptosis, Cardiovascular biology

## Abstract

Mixed-lineage kinase domain-like protein (MLKL) is known as the terminal executor of necroptosis. However, its function outside of necroptosis is still not clear. Herein, we demonstrate that MLKL promotes vascular inflammation by regulating the expression of adhesion molecules ICAM1, VCAM1, and E-selectin in endothelial cells (EC). MLKL deficiency suppresses the expression of these adhesion molecules, thereby reducing EC-leukocyte interaction in vitro and in vivo. Mechanistically, we show that MLKL interacts with RBM6 to promote the mRNA stability of adhesion molecules. In conclusion, this study identified a novel role of MLKL in regulating endothelial adhesion molecule expression and local EC-leukocyte interaction during acute inflammation.

## Introduction

Mixed-lineage kinase domain-like protein (MLKL) is the terminal executor of necroptosis, a form of programmed necrotic cell death, and is controlled by RIPK3 downstream of TNFR activated RIPK1 or TLR3 activated TRIF signaling cascades^1,2^. RIPK3 phosphorylates T357/S358 within the activation loop of MLKL, which leads to a conformational change in MLKL, exposing the N-terminal membrane-disrupting 4-helix bundle domain^1,3,4^. Then, MLKL is further phosphorylated by TAM kinase at Y376 to initiate oligomerization^5^. Ultimately, oligomerized MLKL inserts its N-terminal into the plasma membrane and induces necroptotic cell death^6–8^.

However, recent studies demonstrated that MLKL could exert non-necroptotic functions^9–14^. MLKL controls the degradation of myelin sheath, a specialized membrane of Schwann cell that insulates axon, of injured nerve, subsequently promoting nerve regeneration^9^. Interestingly, the myelin-disrupting activity of MLKL is regulated by the phosphorylation of its S441 residue, but not the canonical RIPK3 phosphorylation sites (S345/S347/T349 in mouse) that occurs during necroptosis. Furthermore, overexpression of the phosphomimetic form of MLKL (S441D) in Schwann cell promotes myelin degradation but has no impact on necroptosis^9^. In addition, several studies have demonstrated that MLKL, instead of inducing necroptosis, controls NLRP3 inflammasome formation and IL-1β release in macrophages upon TLR activation^10–12^. Importantly, upon TNFα stimulation, MLKL regulates the trafficking of endosomes, which contain endocytosed proteins, including TNFR, thus modulating TNFR activation. Furthermore, RIPK3-mediated MLKL phosphorylation promotes the association of MLKL with ESCRT proteins and flotillins, thus enhancing the release of phosphorylated MLKL containing extracellular vesicles or promotes the shedding of MLKL-damaged cellular membrane, which maintains the integrity of cellular membrane and promotes cell survival^13–15^.

Cytoplasmic membrane translocation of MLKL is the hallmark of necroptosis^3,6^. Interestingly, MLKL has been found to quickly translocate to the nucleus in association with RIPK1/RIPK3 in TBZ-treated HT29 cells. This nuclear translocation precedes its translocation to the cytoplasmic membrane and the initiation of necroptosis. Nonetheless, MLKL nuclear translocation is not required for executing necroptosis because MLKL lacking NLS motif could still induce necroptosis^16,17^. However, whether nuclear translocated MLKL regulates the transcription of inflammatory response genes remains unclear.

In the present study, we demonstrate that MLKL regulates the expression of endothelial adhesion molecules in a necroptosis-independent manner. MLKL inhibition or knockdown significantly reduced TNFα-induced inflammatory gene expression, including the expression of adhesion molecules ICAM-1, VCAM-1, and E-selectin, thereby reducing leukocytes adhesion and infiltration in vitro and in vivo. Mechanistically, TNFα stimulation induced the association of MLKL with RBM6, stabilizing the mRNAs of adhesion molecules. Thus, our data revealed a previously unrecognized non-necroptotic function of MLKL in regulating the endothelial inflammatory responses.

## Materials and methods

### Mouse husbandry

The generation of *Mlkl*^−/−^ mice was described previously and only male *Mlkl*^+/+^ mice and *Mlkl*^−/−^ littermates were used in this study. All mice were kept under a specific pathogen-free condition with free access to food and water at the Institute of Interdisciplinary Research Center on Biology and Chemistry. The mouse experiments (Registration No.: IRCBC-2017-004) were approved by the Institutional Animal Care and Use Committee (IACUC) of the Institute of Interdisciplinary Research Center on Biology and Chemistry and performed in accordance with the committee’s guidelines.

### Intravital imaging and quantification of adherent leukocytes

To investigate leukocyte adhesion to the blood vessel upon local inflammatory response in vivo, we adopted a previously described skin flap model^18^. Briefly, the hair on the mouse abdomen was removed with a shaver two days before the experiment. On the day of the experiment, the injection sites on both sides of the abdominal skin were marked by animal tattoo ink. To induce local inflammation, 100 ng of TNFα (50 μl volume) or the same volume of saline was intradermally injected into the marked sites on the left and right abdomen, respectively. After 3.5 h, the mice were deeply anesthetized with an intraperitoneal injection of 10% chloral hydrate at a dose of 5 ml/kg. Then, 100 μl of saline containing 1% FITC-Dextran (2000 kDa molecular weight) and 0.02% rhodamine-6G were injected into the retro-orbital sinus. Thirty minutes after dye injection, two arc-shaped abdominal flaps surrounding the injection site with intact thoracoepigastric artery, vein, and capillaries were prepared. The mice were secured in a prone position, and the skin flaps were gently spread on an imaging thermoplate (Tokai Hit, glass thickness 0.5 mm) with the temperature set to 32 °C. The thermoplate was then mounted on an inverted confocal microscope (Leica SP8). The injection sites were located based on tattoo ink marks. Images were collected with a 20× long-working distance objective (0.7 numerical aperture). Postcapillary venules at the injection sites with a diameter of 30–70 μm and a steady flow were chosen for imaging. Images of both channels were simultaneously recorded at resolution 512 × 100 pixels and four frames per second for 20 s. Five vessels per injection site were imaged. The number of adherent leukocytes, which were rhodamine-6G positive, were counted from the 20 s recording.

### Cells and reagents

Human umbilical vein endothelial cells (HUVEC), human umbilical artery endothelial cells (HUAEC), human dermal microvascular endothelial cells (HDMEC), Endothelial Cell Growth Medium (C-22010), and Endothelial Cell Growth Medium MV (C-22020) were purchased from Promocell. Human and mouse TNFα were purchased from R&D (human TNFα, 210-TA-100/CF; mouse TNFα, 410-MT-500/CF). Necrosulfonamide (NSA) was purchased from Millipore (480073). IKKα/β inhibitor TPCA-1 was obtained from Selleck (S2824). Nec-1s, SM164, and zVAD-fmk were provided by Prof. J. Yuan.

### Adhesion assay

HUVEC were cultured at 2 × 10^4^ cells/well in 48-well plates until confluent, then starved with endothelial basal medium (Promocell, C-22210) containing 0.5% fetal bovine serum (FBS) for 6 h, before treatment with TNFα (100 ng/ml), TNFα (100 ng/ml) + NSA (4 μM), TNFα (100 ng/ml) + TPCA-1 (5 μM) or TNFα (100 ng/ml) + Nec-1s (10 μM) for 12 h. Then, 2 × 10^5^ iBMDM pre-labeled with calcein-AM (4 μM, AAT Bioquest, 22003) were seeded on the HUVEC monolayer and allowed to adhere for 30 min. Non-adherent iBMDM were washed away with 200 μl phosphate-buffered saline (PBS) per well three times, and the number of adherent iBMDM was imaged and quantified.

### Immunofluorescent staining

HUVEC were seeded into 24-well plates containing human fibronectin-coated coverslips and were allowed to grow to confluent. HUVEC were starved with endothelial basal medium containing 0.5% FBS for 6 h, then pretreated with NSA (4 μM), TPCA-1 (5 μM) or Nec-1s (10 μM) for 30 min followed by treatment with 50 ng/ml TNFα for the indicated time. Then, HUVEC were washed twice with PBS and fixed with 4% PFA. Fixed cells were washed twice with PBS and blocked with blocking buffer (PBS containing 3% donkey serum) for 1 h at room temperature. After blocking, HUVEC were stained with rabbit anti-p65 (CST, 8242s), phalloidin-Alexa 568 (Invitrogen, A12380), and imaged with a DMI8 microscope (Leica).

### Cell death analysis

HUVEC lentivirally transduced with RIPK3 or empty vector were cultured in 48-well plates to reach confluence. To induce cell death, HUVEC and HT29 cells were stimulated with TNFα (100 ng/ml), SM164 (100 nM) and zVAD-fmk (25 μM) for the indicated times. PI (1 μg/ml, Sigma, P4170) and Hoechst 33342 (2 μM, Life technology, H13399) diluted in endothelial basal medium containing 0.5% FBS were added to HUVEC before incubation (37 °C, 5% CO_2_) for 10 min. Cells were imaged with an SP8 confocal microscope (Leica).

### MEF isolation

*Mlkl*^+/+^ and *Mlkl*^−/−^ mouse embryos (12.5 p.c.) were freshly dissected and minced after removing the heart, liver, and other visible internal organs. Then, 10 ml of 0.25% trypsin was added per embryo and incubated at 37 °C for 20 min to produce single-cell suspensions. Suspensions were transferred into 10 cm dish containing 10 ml DMEM complete medium (DMEM + 10% FBS + 1% penicillin–streptomycin solution) and incubated (37 °C, 5% CO_2_) for expansion. After expansion, aliquots were frozen and stored in liquid nitrogen.

### Analysis of retinal vasculature

Adult and P6 pups of *Mlkl*^+/+^ and *Mlkl*^−/−^ littermates were sacrificed. The eyeballs were isolated and fixed with 4% PFA for 8 h. Retinas were dissected and blocked with 3% normal donkey serum in PBS-T for 1 h at RT, before staining with hamster anti-CD31 (ThermoFisher Scientific, MA3105) and rabbit anti-Desmin (Abcam, ab15200) overnight at 4 °C. Retinas were imaged with an SP8 confocal microscope (Leica).

### Western blot analysis

Cell lysates were separated on 10% SDS-PAGE gels and transferred onto nitrocellulose membranes. The membranes were blocked with 5% bovine serum albumin (BSA) and incubated with indicated primary antibodies at 4 °C for 12 h. After washing three times with TBS-T, membranes were incubated with secondary antibodies at RT for 1 h. ECL western blot substrate was used to visualize protein bands. Primary and secondary antibodies: rabbit anti-MLKL (Proteintech, 21066-1-AP), rabbit anti-p-MLKL (T357/S358) (Abcam, ab187091), rabbit anti-RIPK1 (CST, 8737), rabbit anti-RIPK3 (Abcam, ab72106), rabbit anti-ICAM1 (CST, 4915S), rabbit anti-VCAM1 (CST, 13662S), mouse anti-GAPDH (TransGen, HC301-02), mouse anti-Tubulin (TransGen, HC101-02), horseradish peroxidase (HRP) conjugated donkey anti-rabbit and HRP conjugated donkey anti-mouse were obtained from Jackson lab.

### Proximity ligation assay

HUVEC were fixed with 4% PFA for 15 min at room temperature. Fixed cells were blocked with Duolink Blocking Buffer (Sigma, Duo92002) for 1 h at room temperature and incubated with rabbit anti-MLKL and mouse anti-RBM6 antibodies overnight. Duolink In Situ proximity ligation assay (PLA) probe anti-rabbit PLUS IgG (Sigma, Duo92002) and anti-mouse MINUS IgG (Sigma, Duo92004) were added to the cells, and cells were incubated for 1 h at 37 °C. Duolink In Situ Detection Reagent Red (Sigma, Duo92008) was used for the detection of PLA signal. Ligation reagents containing ligase were added, and cells were incubated for 30 min at 37 °C. Afterwards, amplification reagents containing polymerase were added, and cells were incubated for 90 min at 37 °C. The PLA signal was observed as red pots by Leica SP8 confocal microscope and was analyzed using Image J.

### liquid chromatography-mass spectrometry (LC-MS/MS) sample preparation and analysis

PBS or TNFα-treated HUVEC were lysed in radioimmunoprecipitation assay (RIPA) buffer. Cell lysates were then incubated with rabbit anti-MLKL (GeneTex, GTX107538) and anti-rabbit Dynabeads (Invitrogen, 00695229) overnight at 4 °C. The immunoprecipitated proteins were re-dissolved in a buffer consisting of 8 M urea and 100 mM Tris·HCl (pH 8.5). Disulfide bridges were reduced by adding Tris (2-carboxyethyl) phosphine (TCEP) to a final concentration of 5 mM and incubating for 20 min. Reduced cysteine residues were alkylated by adding iodoacetamide (IAM) to 10 mM and incubating for 15 min in the dark at room temperature. Three volumes of buffer containing 100 mM Tris, pH 8.5, 1 mM CaCl_2_ was added to reduce the urea concentration to 2 M. The protein mixture was then digested with trypsin (Promega, enzyme-to-substrate ratio of 1:50 [w/w]) overnight n at 37 °C. Subsequently, the peptides were collected by centrifuging and desalted with C18 StageTips. The peptide mixture was analyzed using an on-line EASY-nL-LC 1000 coupled with an Orbitrap Fusion mass spectrometer. The samples were loaded into 15 cm homemade capillary columns (C18-AQ, 1.9 mm, Dr.Maisch, 100 mm I.D.). Mobile phase A consisted of 0.1% FA, 2% ACN, and 98% H_2_O, and mobile phase B consisted of 0.1% FA, 2% H_2_O, and 98% ACN. A 60 min gradient (mobile phase B: 3% at 0 min, 8% at 5 min, 22% at 40 min, 35% at 51 min, 40% at 55 min, 95% at 58 min, 95% at 60 min) was used at a static flow rate of 300 nl/min. The data were acquired in a data-dependent (top-20) mode. For MS1, the scan range was set to 300–1800 *m*/*z* at a resolution of 120,000. The AGC target was set as 5e6 with a maximum injection time of 50 ms. For MS2, the resolution was set to 30,000 with a fixed first mass of 120 *m*/*z*. The AGC target was set to 5e5 and the maximum injection time was set to 50 ms. The MS raw data files were submitted to the Maxquant search engine (version 1.5.2.8). The canonical human protein database (downloaded from the UniProt database on September 2016) was used for database searching. The precursor mass tolerance was set to 10 ppm, and the fragment mass tolerance was 0.6 Da. The protein N-terminal acetylation (+42.010565 Da) and oxidation on methionine (+15.994915 Da, M) were set as dynamic modifications and carbamidomethyl on cysteine (+57.021464 Da, C) was set as fixed modification. Trypsin was used as the enzyme, and up to 1 missed cleavage site was allowed. The extracted ion chromatographs (XICs) were used for label-free quantification. Match between run was used for better quantification among different samples. The abundance of a protein was calculated by summing all quantified razor peptides.

### RNA sequencing and bioinformatic analysis

Total RNAs of HUVEC treated with vehicle, TNFα, NSA, or TNFα and NSA were extracted using the Trizol and UNlQ-10 column Trizol total RNA isolation kit (Sangon, 511321) according to the manufacturer’s instructions. Total RNA samples were processed using VAHTS Stranded mRNA-seq Library Prep Kit for Illumina (Vazyme Biotech Co., NR-602). Briefly, the polyA fraction (mRNA) was purified using mRNA capture beads from 1 µg of total RNA input followed by the generation of double-stranded cDNA and fragmentation. The library purification was then processed with AMPure XP beads to eliminated the oligo dimers and short fragments. For each library, the concentration was determined using a Qubit (ThermoFisher Scientific) and qPCR (Vazyme Biotech Co., NQ-103), and the size distribution was evaluated using an Agilent Bioanalyzer 2100 (Agilent Technologies). The sequencing was performed by using the single-index sequencing primers on an Illumina Hiseq X Ten instrument following the 150-bp paired-end sequencing procedure. The raw sequencing data were first filtered by FASTQC (v0.11.8) and fastp (v0.19.4) with default software parameters. After filtering, clean reads were aligned to the GRCh38 genome using STAR (v2.7.2b), and the output BAM files were sorted and de-duplicated with samtools (v1.9). The gene expression count matrix was then collected by featureCounts (v1.6.4). Further analysis was performed using R (v3.6.1) and Bioconductor (v3.09) packages. All the annotations were done through biomaRt (Ensembl release 95). Principle component analysis (PCA) and sample-to-sample correlation were performed to profile the overall sample clustering. All samples had no obvious deviation and were kept for further analysis. Differentially expressed genes (DEGs) were defined by a fold change of >1.5 and an adjusted *p* value of <0.05 using DESeq2 (v1.22.2). Gene Ontology (GO) biological process enrichment analysis was performed using clusterProfiler (v3.10.1) with the default settings.

### Real-time PCR (qPCR) analysis

Total RNA was extracted using the Trizol and UNlQ-10 column Trizol total RNA isolation kit (Sangon, 511321) according to the manufacturer’s instructions. RNA was transcribed into cDNA using the ReverTra Ace® qPCR RT master mix with gDNA remover (Toyobo, FSQ-301). Real-time PCR was performed using the PowerUp SYBR Green mix (Life technology, A25778). Gene expression levels were calculated based on the Delta-Delta Ct relative quantification methods. Primers used in this study are listed in Supplementary Table S2.

### RNA decay assay

HUVEC were first lentivirally transduced with shCtrl or shRBM6, then seeded into 12-well plates allow to reach confluence. HUVEC were treated with starvation medium (endothelial basal medium supplemented with 0.5% FBS) for 6 h, followed by TNFα (50 ng/ml) treatment. Afterward, actinomycin-D (5 μM, MCE, 50-76-0) diluted in the starvation medium was added to HUVEC culture. HUVEC were harvested 2, 4 and 6 h after actinomycin-D treatment. Total RNA was isolated and cDNA was analyzed by real-time PCR. *ICAM1, VCAM1 and E-selectin* mRNA levels were normalized to 18S rRNA and shown as percentage of remaining mRNA relative to time 0. Regression analysis used a two-rate exponential decay.

### Statistics

All data are presented as mean ± SD. A two-tailed Student’s *t* test was used for the comparison between two experimental groups. Values of *P* < 0.05 were considered statistically significant.

## Results

### HUVEC is insensitive to RIPK3-mediated necroptosis

**Fig. 1:**
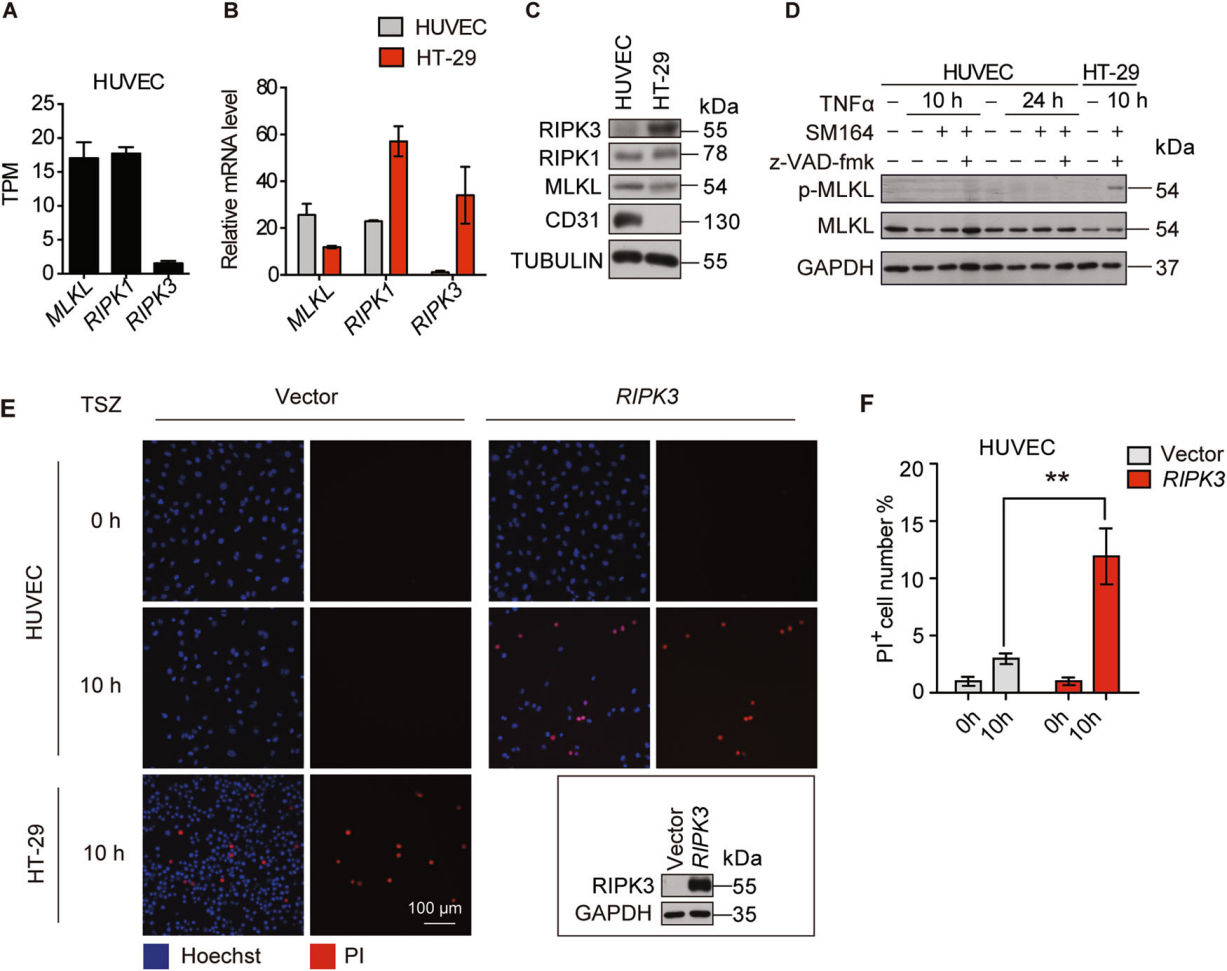
HUVEC express low levels of RIPK3 and are insensitive to TNFα/SM164/z-VAD-fmk induced necroptosis. **a** RNA-seq analysis of RIPK1, RIPK3, MLKL in HUVEC expressed as transcripts per million (TPM) (*n* = 3 biological triplicates, mean ± SD). **b**, **c** Real-time PCR (**b**) and immunoblot (**c**) validation of the expression of RIPK1, RIPK3, and MLKL in HUVEC and HT-29. **d** Expression of p-MLKL (T357/S358) and total MLKL, and GAPDH in HUVEC and HT-29 cells treated with TNFα (100 ng/ml), SM164 (100 nM) and z-VAD-fmk (25 μM). **e**, **f** Vector control or RIPK3 overexpressed HUVEC were treated with TSZ for 10 h and necroptotic cells were visualized by PI staining. HT29 served as a positive control for TSZ-induced necroptosis. Quantification of PI-positive cells is shown in (F). Data are representative of three independent experiments, with each experiment containing biological triplicates. Mean ± SD; **P < 0.01; Student’s *t* test.

To investigate the role of RIPK1-RIPK3-MLKL in endothelial cells, we first determined their expression levels. RNA-seq analysis of HUVEC demonstrated that MLKL and RIPK1 were highly expressed in HUVEC, whereas RIPK3 was lowly expressed (Fig. 1a). Real-time PCR analysis also demonstrated a similar result (Supplementary Fig. S1A). Comparison of their expression in HUVEC and HT-29, a human colorectal carcinoma cell line, revealed that RIPK1 and MLKL were expressed in HUVEC and HT29, while RIPK3 was normally expressed in HT29 but at an extremely low level in HUVEC (Fig. 1b, c). Especially, the expression of RIPK3 in HUVEC could not be induced by TNFα (Supplementary Fig. S1B). In addition, analysis with HUVEC, HUAEC, HDMEC, and SV40 large T-antigen immortalized HDMEC (iHDMEC) demonstrated that RIPK3 was expressed at very low levels in all those endothelial cells (Supplementary Fig. S1C). The necroptotic activity of MLKL depends on the phosphorylation of its T357/S358 residues by RIPK3, and the protein level of RIPK3 is correlated with the incidence of necroptosis. Therefore, we tested whether MLKL could induce necroptosis in HUVEC by treating the cells with TNFα, Smac-mimetic SM164, and pan-caspase inhibitor z-VAD-fmk (TSZ), a well-established combination to induce necroptosis. TSZ treatment-induced MLKL phosphorylation at T357/S358, consequently, triggered necroptotic cell death in HT29 within 10 h (Fig. 1d, e). On the contrary, TSZ did not induce MLKL T357/S358 phosphorylation and necroptotic cell death in HUVEC, even after prolonged TSZ treatment for 24 h (Figs. 1d, e and S1D). Interestingly, TSZ was able to induce necroptosis in HUVEC when RIPK3 expression was reconstituted (Fig. 1e, f). Thus, these data clearly indicate that HUVEC is insensitive to TSZ-induced necroptosis due to the low expression of RIPK3.

### MLKL controls adhesion molecules expression

**Fig. 2:**
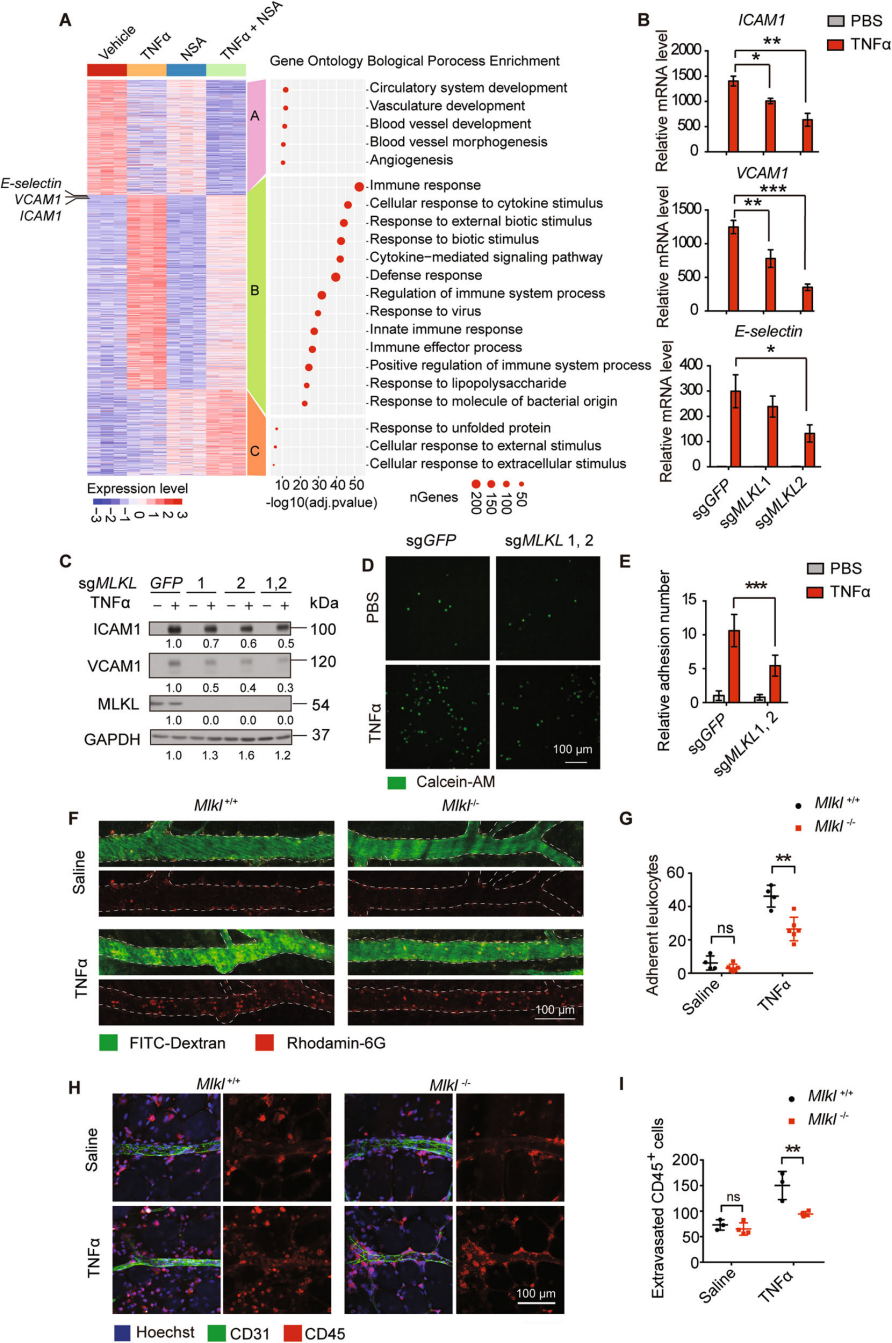
MLKL regulates adhesion molecules expression as well as EC-leukocyte interaction. **a** Heatmap of normalized expression dynamics of top 2000 DEGs in vehicle and TNFα, NSA, or TNFα + NSA treated cells. K-means clustering analysis identified three clusters of DEG according to their expression patterns. Left panel shows the gene ontology biological process analysis of the three DEG clusters (*n* = 3 biological triplicates). **b**, **c** Control or MLKL knockout HUVEC were stimulated with TNFα (50 ng/ml) for 6 h. Expression levels of adhesion molecules were determined by real-time PCR (**b**) and immunoblot (**c**). **d**, **e** Control or MLKL knockout HUVEC monolayers were pretreated with TNFα (100 ng/ml) for 12 h, then incubated with calcein-AM-labeled iBMDM for 30 min and adherent iBMDM were imaged (**d**) and quantitated (**e**). Calcein-AM, green. Scale bar, 100 μm. **f**, **g** *Mlkl*^*+/+*^ (*n* = 3–4) and *Mlkl*^*−/*^^−^ (*n* = 3–6) mice were intradermally injected with 100 ng TNFα (in 50 μl saline) into the right side of abdominal skin to induce local inflammation, with the same volume of saline injected into the left side as control. Four hours later, the leukocytes (rhodamine-6G^+^) that adhered to the vessel wall were imaged by intravital confocal microscopy (**f**) and quantitated (**g**). Vessel were visualized with FITC-Dextran. **h**, **i** Eight hours after TNFα injection, the extravasated leukocytes were imaged (**h**) and quantitated (**i**). Leukocytes were stained with CD45 antibody and endothelial cells were stained with CD31 antibody. Data shown in Scale bar, 100 μm. Data (**b**–**e**) are representative of three independent experiments, with each experiment containing biological triplicates. Mean ± SD; *P < 0.05, **P < 0.01, ***P < 0.001; ns, not significant; Student’s *t* test.

To explore whether MLKL plays a role in the endothelial inflammatory response, HUVEC were pretreated with necrosulfonamide (NSA)^1^, an MLKL specific inhibitor, followed by TNFα stimulation, then the HUVEC were subjected to whole transcriptomic RNA-seq analysis. K-means clustering of the differentially expressed genes (DEG) revealed that TNFα stimulation caused a dramatic change in gene expression in HUVEC (Figs. 2a, S2A, Supplementary Table S1). Notably, TNFα suppressed the expression of genes involved in angiogenesis and vascular development (Fig. 2a, Cluster A), but potently upregulated the expression of genes that participate in inflammatory responses, including cytokines, chemokines, and adhesion molecules *ICAM1, VCAM1, and E-selectin* (Fig. 2a, Cluster B). Interestingly, NSA treatment significantly reduced the expression of those TNFα-induced inflammatory-related genes (Fig. 2a, Cluster B). Endothelial cells function as an important barrier between the inflamed tissue and the circulating immune cells, with upregulated cytokines and chemokines promoting the recruitment of leukocytes, while the increased adhesion molecules on the endothelial surface facilitate leukocyte adhesion and extravasation. Consequently, extravasated leukocytes clear the microbes or cell debris, thereby promoting resolution of inflammation. However, uncontrolled immune cell recruitment and infiltration can have the opposite effects and cause tissue damage^19^. As endothelial adhesion molecules determine leukocytes adhesion/extravasation, and the EC-leukocyte interaction can be precisely evaluated both in vitro and in vivo, we, therefore, mainly focused on investigating how MLKL affects the expression of endothelial adhesion molecules during inflammation.

Adhesion molecules such as *ICAM1*, *VCAM1*, and *E-selectin* were expressed at very low levels in resting EC, but their expression could be quickly induced upon TNFα stimulation, peaking at approximately 6 h post stimulation (Supplementary Fig. S2B). Real-time qPCR analysis of HUVEC lysates confirmed that NSA strongly inhibited TNFα-induced *ICAM1*, *VCAM1*, and *E-selectin* expression (Supplementary Fig. S2C). Similarly, sgRNA-mediated MLKL deletion potently reduced both mRNA and protein levels of ICAM1, VCAM1, and E-selectin in HUVEC (Fig. 2b, c). We further validated the role of MLKL in regulating the expression of *Icam1*, a broadly expressed adhesion molecule, in mouse embryonic fibroblasts (MEF) and found that its expression in TNFα-stimulated *Mlkl*^*−/−*^ MEF was dramatically reduced compared to *Mlkl*^*+/+*^ MEF (Supplementary Fig. S3A, B). It is known that human MLKL cannot induce necroptosis in mouse cells^20,21^. However, the reconstitution of both human MLKL and murine MLKL in *Mlkl*^*−/−*^ MEF successfully restored *Icam1* expression (Supplementary Fig. S3C, D), implying that MLKL regulates *Icam1* expression independent of its necroptosis-executing function. Thus, our data demonstrated that MLKL regulates TNFα-induced adhesion molecule expression in a necroptosis-independent manner.

### MLKL controls EC-leukocyte interaction in vitro and in vivo

Next, we examined if MLKL deficiency impaired EC-leukocyte interaction during inflammation. To this end, we first evaluated the effects of MLKL on EC-leukocyte adhesion in a static adhesion assay. Calcein-AM labeled bone marrow-derived monocytes (BMDMs) were incubated with PBS- or TNFα-treated HUVEC monolayers for 30 min. Then non-adherent BMDMs were washed away, and the remaining adherent BMDMs on the HUVEC monolayers were quantified, showing that ten-fold more BMDMs adhered to TNFα-treated HUVEC monolayers compared to the PBS-treated group. However, MLKL knockout in HUVEC dramatically reduced the number of adherent BMDMs due to reduced adhesion molecule expression by MLKL deficiency (Fig. 2d, e).

To further validate this finding in vivo, we subcutaneously injected 100 ng of TNFα into the abdomen skin of *Mlkl*^*+/+*^ littermates and *Mlkl*^*−/−*^ mice, which are viable and healthy and show no defects in vascular development (Supplementary Fig. S4), to induce a local inflammatory response. The advantage of this model was that strong EC inflammation could be induced at a defined region with minimal effect on circulating immune cells compared to the widespread activation of circulating immune cells in systemic inflammation models induced by tail vein TNFα injection, thus allowed us to precisely evaluate the contribution of endothelial MLKL to EC-immune cell interaction (Supplementary Fig. S5A). Intravital imaging analysis revealed that local injection of TNFα dramatically increased the number of adhered rhodamine-6G positive leukocytes on the wall of postcapillary venules. However, the number of adhered leukocytes in the *Mlkl*^*−/−*^ mice was significantly reduced compared to that in their *Mlkl*^*+/+*^ littermates (Fig. 2f, g). In another independent experiment, we excised the skin 8 h after TNFα injection and analyzed extravasated leukocytes by CD45 staining. As a result of reduced leukocyte adhesion, the number of extravasated leukocytes in *Mlkl*^*−/−*^ mice was also significantly reduced compared to those in WT littermate controls (Fig. 2h, i).

To corroborate that the reduced leukocyte adhesion in *Mlkl*^*−/−*^ mice was caused by the reduction of ICAM1, VCAM1, and E-selectin in EC, not the reduction of their corresponding ligands and receptors expressed in leukocytes, we analyzed the expression of ICAM1 receptors CD18 and CD11b, VCAM1 receptor CD49d, and E-selectin ligand CD162 in peripheral leukocytes. FACS analysis showed that the percentage of CD18, CD11b, CD49d, and CD162 expressing leukocytes was similar in both *Mlkl*^*+/+*^ and *Mlkl*^*−/−*^ littermates (Supplementary Fig. S5B, C). Additional real-time PCR analysis demonstrated that MLKL deficiency did not affect the expression levels of CD18, CD11b, CD49d, and CD162 in peripheral leukocytes (Supplementary Fig. S5D). We further isolated peripheral leukocytes from *Mlkl*^*+/+*^ and *Mlkl*^*−/−*^ mice and measured their adhesion to control or TNFα-activated HUVEC monolayers, showing that both *Mlkl*^*+/+*^ and *Mlkl*^*−/−*^ leukocytes adhered to TNFα-activated EC monolayers at similar levels (Supplementary Fig. S5E, F). Thus, our in vitro and in vivo data suggests that reduced leukocytes adhesion in *Mlkl*^*−/−*^ mice is solely due to the reduction of adhesion molecules in *Mlkl*^*−/−*^ EC.

### MLKL regulates adhesion molecule expression independent of RIPK1 kinase activity and RIPK1 scaffolding function-mediated NF-κB activation

**Fig. 3:**
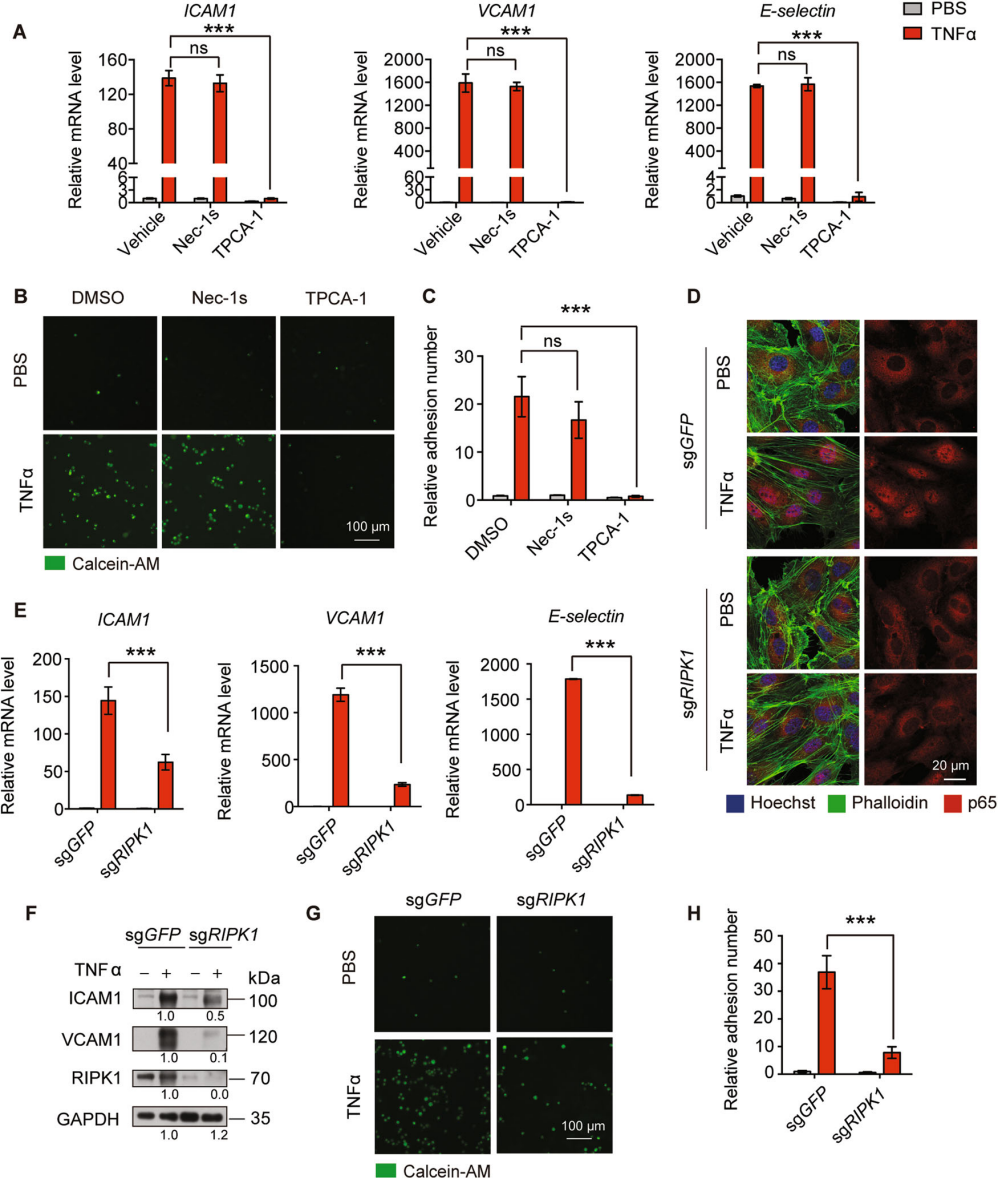
MLKL regulates adhesion molecules expression is independent of RIPK1 kinase activity and RIPK1 mediated NF-κB activation. **a** HUVEC were pretreated with Nec-1s (10 μM) or TPCA-1 (5 μM) for 1 h then were stimulated with TNFα (100 ng/ml) for 6 h. The expression levels of *ICAM1*, *VCAM1*, and *E-selectin* were determined by real-time PCR. **b**, **c** HUVEC were treated with Nec-1s (10 μM) or TPCA-1 (5 μM) for 1 h followed by TNFα (100 ng/ml) stimulation for 12 h and incubated with calcein-AM-labeled iBMDM for 30 min, then adherent iBMDM were imaged (**b**) and quantitated (**c**). Scale bar, 100 μm. **d** Control or RIPK1 knockout HUVEC were stimulated with TNFα (50 ng/ml) for 15 min and nuclear translocation of p65 was determined by immunofluorescence staining. Scale bar, 20 μm. **e**, **f** Control or RIPK1 knockout HUVEC were stimulated with TNFα (50 ng/ml) for 6 h and then expression levels of adhesion molecules was determined by real-time PCR (**e**) and immunoblot (**f**). **g**, **h** Control or RIPK1 knockout HUVEC monolayers were first stimulated with TNFα (100 ng/ml) for 12 h, then incubated with calcein-AM-labeled iBMDM for 30 min and adherent iBMDM were imaged (**g**) and quantitated (**h**). Data are representative of three independent experiments, with each experiment containing biological triplicates. Mean ± SD; ***P < 0.001; ns, not significant; Student’s *t* test. Scale bar, 100 μm.

Though RIPK3 was not present, RIPK1 was highly expressed in HUVEC (Fig. 1a). It has been shown that RIPK1 kinase activity, but not RIPK3, is needed for MLKL mediating hepatocyte necroptosis in autoimmune hepatitis^22^. Thus, we asked whether RIPK1 kinase activity is required for MLKL-regulated adhesion molecules expression. To this end, HUVEC were pretreated with RIPK1 kinase inhibitor Nec-1s before TNFα stimulation^23^. It is well established that the NF-*κ*B pathway controls *ICAM1*, and *VCAM1* mRNA transcription^24,25^; therefore, HUVEC were pretreated with IKKα/β inhibitor TPCA-1 as a positive control. Consistent with previous reports, real-time PCR analysis showed that TPCA-1 pretreatment completely abolished TNFα-induced *ICAM1, VCAM1, and E-selectin* mRNA expression in HUVEC. In contrast, Nec-1s did not affect *ICAM1, VCAM1*, and *E-selectin* expression in HUVEC (Fig. 3a). Similarly, Nec-1s pretreatment had no effect on TNFα-induced *Icam1* expression in MEF (Supplementary Fig. S6A). Furthermore, the static adhesion experiment showed that TPCA-1, but not Nec-1s, strongly reduced the number of BMDMs adhering to HUVEC monolayers (Fig. 3b, c). Thus, our data demonstrate that RIPK1 kinase activity is not involved in the regulation of adhesion molecule expression.

RIPK1 kinase activity is required for the induction of apoptosis and necroptosis. However, independent of its kinase activity, polyubiquitinated RIPK1 also functions as an important scaffold protein, which recruits NF-κB essential modulator (NEMO) and activates the NF-κB pathway to initiate downstream inflammatory and pro-survival programs^26–28^. As expected, RIPK1 depletion abolished TNFα-induced NF-κB activation in HUVEC, as evidenced by a dramatic reduction of p65 nuclear translocation (Fig. 3d). Consequently, mRNA and protein levels of ICAM1, VCAM1, and E-selectin upon TNFα stimulation were reduced in RIPK1-deficient HUVEC (Fig. 3e, f), which eventually reduced the EC-leukocyte interaction (Fig. 3g, h). Consistently, RIPK1 deficiency also impaired TNFα-induce *Icam1* expression in MEF (Supplementary Fig. S6B, C).

To test whether MLKL crosstalk with the NF-*κ*B pathway in regulating adhesion molecule expression, we examined the interaction between MLKL and TNFR1, RIPK1, and downstream signaling transducers. However, immunoprecipitation experiments showed that MLKL could not be recruited to the TNFR signaling complex and IKK complex in the early stage of TNFα signaling (Supplementary Fig. S7A, B). We also confirmed that that MLKL did not interact with RIPK1, NEMO, IKKα, and p65 regardless of TNFα stimulation (Supplementary Fig. S7C). Importantly, unlike RIPK1 deficiency abrogating p65 nuclear translocation (Fig. 3d), MLKL depletion did not alter TNFα-induced p65 nuclear translocation in HUVEC (Supplementary Fig. S7D). Collectively, our data demonstrated that RIPK1 scaffolding function-mediated NF-κB activation is not involved in MLKL-regulated *ICAM1*, *VCAM1*, and *E-selectin* expression.

### MLKL nuclear translocation promotes adhesion molecules expression

**Fig. 4:**
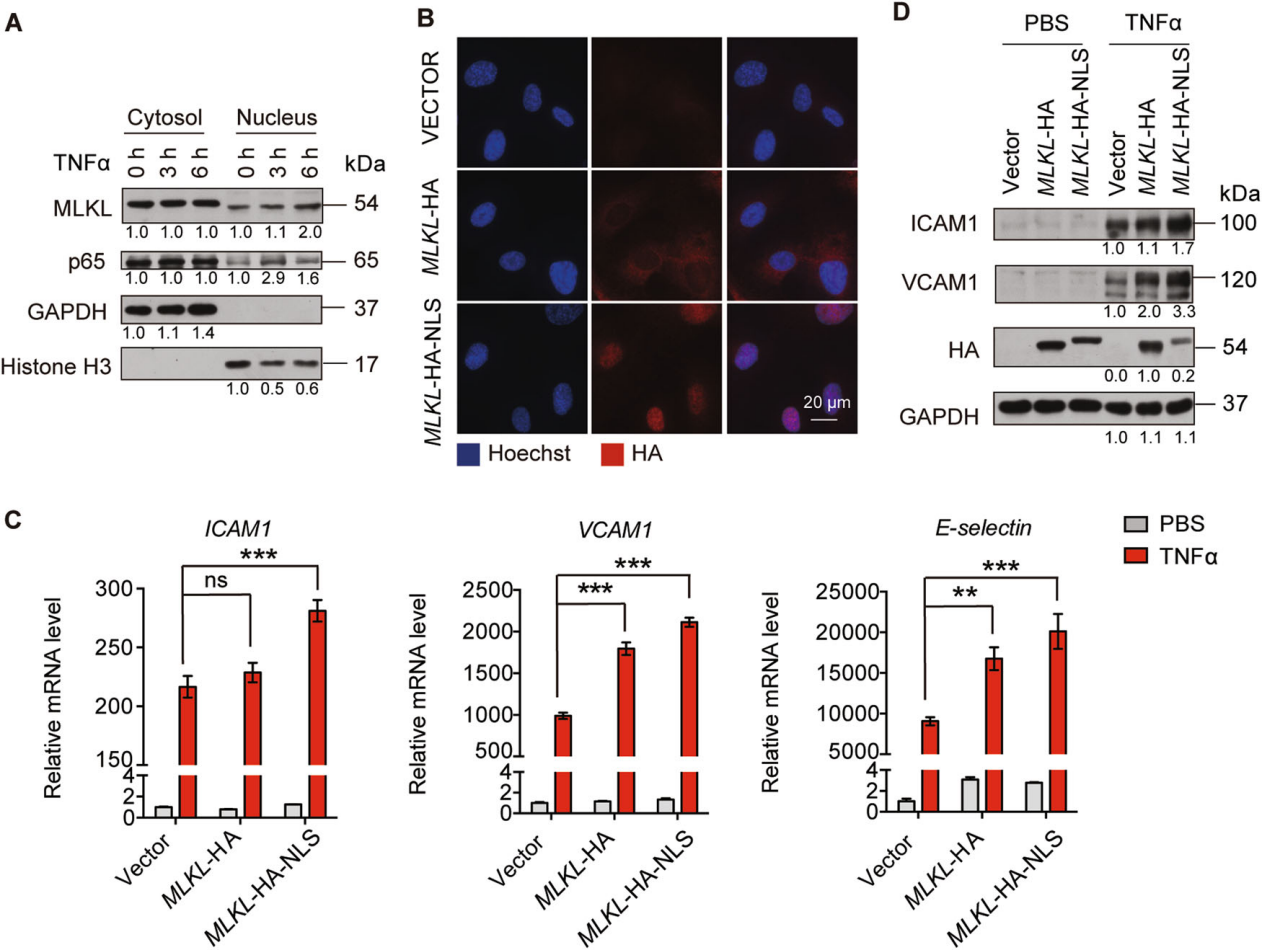
MLKL nuclear translocation promotes adhesion molecules expression. **a** HUVEC were treated with TNFα (50 ng/ml) for the indicated times, then MLKL content in the cytoplasmic and nuclear fraction was determined by immunoblot. **b**–**d** HUVEC were lentivirally transduced with MLKL-HA and MLKL-HA-NLS followed by TNFα (50 ng/ml) treatment for 6 h, before the expression and localization of MLKL-HA or MLKL-HA-NLS in HUVEC was confirmed by immunofluorescent staining against HA tag (**b**). The expression of *ICAM1*, *VCAM1*, and *E-selectin* was determined by real-time PCR (**c**) and immunoblot (**d**). Data are representative of three independent experiments, with each experiment containing biological triplicates. Mean ± SD; **P < 0.01, ***P < 0.001; ns, not significant; Student’s *t* test.

The previous study demonstrated that MLKL quickly translocates to the nucleus before executing necroptosis^16^, suggesting that it might modulate the function of some nuclear-localized proteins or influence the expression of inflammatory response genes. This raise the question whether its nuclear translocation plays a role in regulating adhesion molecules expression. To this end, we first analyzed MLKL localization in HUVEC by fractionation, showing that MLKL mainly localized in the cytoplasm of HUVEC. However, a small fraction of MLKL did localize in the nucleus, which interestingly, increased over time upon TNFα stimulation (Fig. 4a). To further decipher the effect of MLKL nuclear translocation on adhesion molecule expression in endothelial cells, we expressed nuclear-localized MLKL by adding an additional NLS to its C-terminal (MLKL-HA-NLS). Immunofluorescent staining confirmed the nuclear localization of MLKL-HA-NLS, while MLKL-HA was mainly localized in the cytoplasm (Fig. 4b). MLKL-HA overexpression increased TNFα-induced adhesion molecule expression in HUVEC. Notably, MLKL-HA-NLS was more potent than MLKL-HA in potentiating ICAM1, VCAM1, and E-selectin expression, indicating that nuclear translocation of MLKL had a positive impact on adhesion molecule expression (Fig. 4c, d).

### MLKL interacts with RBM6 in regulating the mRNA stability of adhesion molecules

**Fig. 5:**
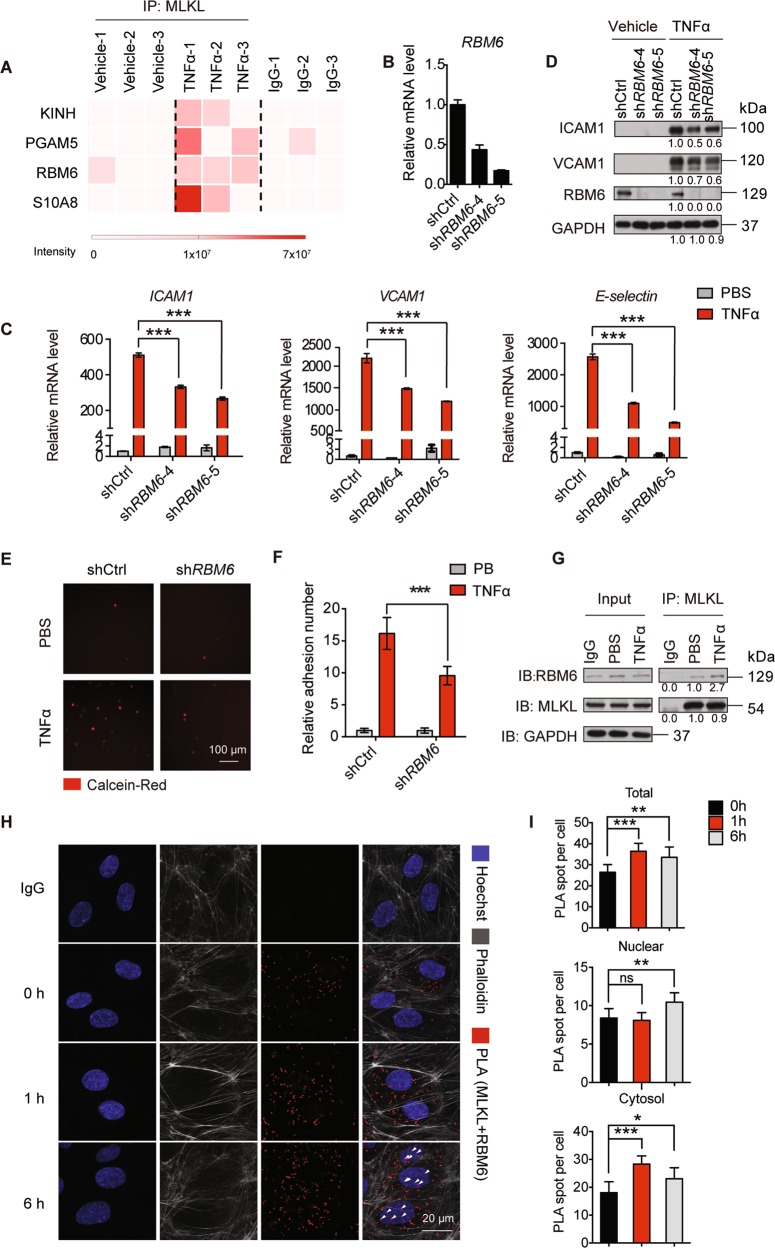
MLKL interacts with RBM6. **a** Heatmap showing the intensity of potential MLKL-interacting proteins identified by mass spectrometry (*n* = 3 biological replicates). **b** HUVEC were transduced with shCtrl or shRBM6 and the knockdown efficiency was validated by real-time PCR. **c**, **d** The expression of adhesion molecules in shCtrl or shRBM6 transduced HUVEC post TNFα (50 ng/ml) stimulation was determined by real-time PCR (**c**) and immunoblot (**d**). **e**, **f** Control or RBM6 shRNA transduced HUVEC monolayers were first stimulated with TNFα (100 ng/ml) for 12 h, then incubated with calcein-Red-labeled iBMDM for 30 min and adherent iBMDM were imaged (**e**) and quantitated (**f**). Scale bar, 100 μm. **g**–**i** The interaction of MLKL and RBM6 was confirmed by immunoprecipitation (**g**), and PLA (**h**). PLA spots were quantitated in (**i**). Arrowheads indicated the nuclear PLA signal. Hoechst, blue; Phalloidin, gray; PLA (MLKL and RBM6), red. Data are representative of three independent experiments, with each experiment containing biological triplicates. Mean ± SD; *P < 0.05, **P < 0.01, ***P < 0.001; ns, not significant; Student’s *t* test. Scale bar, 20 μm.

The N-terminal of MLKL mediates cytoplasmic membrane disruption, and its C-terminal pseudokinase domain contains an NLS motif that is required for nuclear shuttling^16^. However, none of these domains has been demonstrated to possess DNA- or RNA-binding ability. Thus, we hypothesized that MLKL might regulate gene expression by interacting with other proteins. To this end, we immunoprecipitated endogenous MLKL from control or TNFα-treated HUVEC and performed mass spectrometry (MS) analysis. The most highly enriched proteins in TNFα treated HUVEC were Kif5B, PGAM5, RNA-binding motif protein 6 (RBM6), and S10A8 (Fig. 5a). To determine which protein participates in MLKL-regulated adhesion molecule expression, we performed shRNA-based knockdown in HUVEC and measured the expression of adhesion molecules. Results demonstrated that only RBM6 knockdown markedly reduced *ICAM1* and *VCAM1* mRNA, and protein levels in TNFα-treated HUVEC (Fig. 5b–d). In contrast, knockdown of PGAM5, Kif5B, and S10A8 did not affect ICAM1 and VCAM1 protein levels (Supplementary Fig. S8A–C). Furthermore, the static adhesion assay confirmed that knockdown of RBM6 in HUVEC significantly reduced BMDM adherence as a consequence of decreased adhesion molecule expression (Fig. 5e, f).

To understand the spatiotemporal dynamics of the MLKL and RBM6 interaction during inflammation, we first analyzed the spatial distribution of MLKL and RBM6 in HUVEC. Consistent with previous reports (*16, 29*), the majority of HA-MLKL and Flag-RBM6 were expressed in the cytosol and nucleus, respectively. However, weak expression signals of nuclear MLKL and cytosolic RBM6 could be observed irrespective of TNFα stimulation (Supplementary Fig. S9). Next, a co-immunoprecipitation experiment was performed to validate the endogenous MLKL-RBM6 interaction. MLKL interacted with RBM6 to some degree in unstimulated HUVEC, and their interaction was increased upon TNFα stimulation (Fig. 5g). To further investigate the endogenous MLKL-RBM6 interaction in a spatially resolved manner, a proximity ligation assay (PLA)-based spatial analysis was adopted. PLA revealed that the total amount of MLKL and RBM6 in close proximity was increased in HUVEC upon TNFα treatment. Interestingly, the MLKL and RBM6 interaction in the cytosol first increased one-hour after TNFα stimulation and then decreased to normal, while their interaction in the nucleus increased six hours after TNFα stimulation (Fig. 5h, i). This is likely a consequence of MLKL nuclear translocation upon TNFα stimulation.

**Fig. 6:**
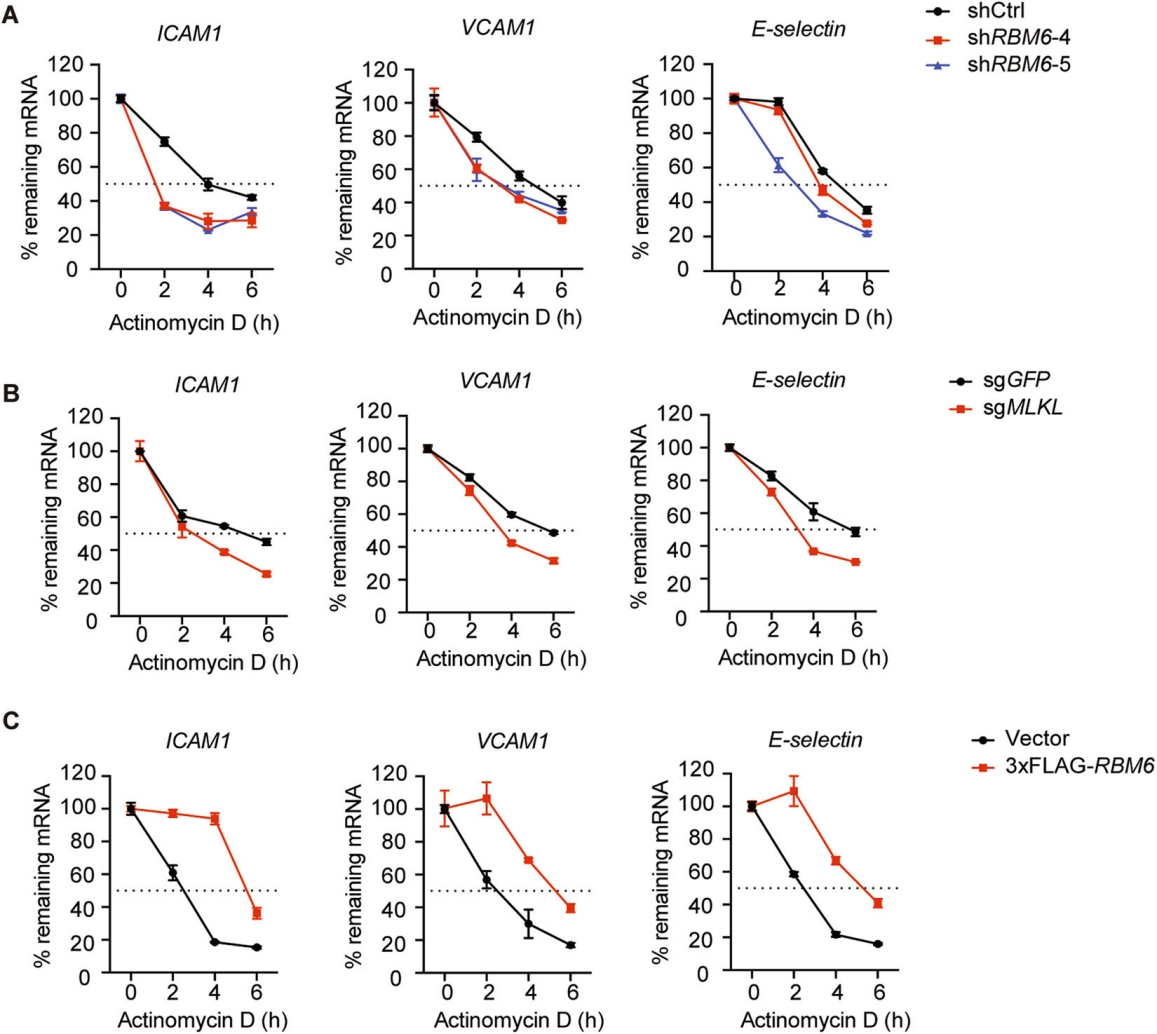
MLKL interacts with RBM6 in regulating the stability of adhesion molecule mRNA. **a**–**c** HUVEC lentivirally transduced with control or RBM6 shRNA (**a**), vector or 3xflag-RBM6 (**b**) and control or MLKL sgRNA were (**c**) pretreated with TNFα (50 ng/ml), then incubated with actinomycin-D (5 μM). Cells were harvested at the indicated times and total RNA was isolated and analyzed, with mRNA levels of adhesion molecules normalized to 18S RNA and expressed as percentage of remaining mRNA relative to time 0. Data are representative of two independent experiments, with each experiment containing biological triplicates. Mean ± SD. Regression analysis used a two-rate exponential decay model.

RBM6 possesses multiple RNA-binding motifs. It belongs to a large RNA binding protein family that plays an essential role in almost every aspect of RNA metabolism, including RNA splicing, and stability^29,30^. A few studies have implicated that RBM6 functions as a tumor suppressor and controls tumor growth by regulating RNA alternative splicing in tumors^31,32^; however, its function remains largely unexplored. Analysis of RNA-seq data revealed that MLKL inhibition by NSA suppressed the expression of numerous inflammatory-related genes (Fig. 2a) but did not cause any apparent changes in the global alternative splicing landscape (Supplementary Fig. S10A). Besides, alternative protein coding usage analysis of *ICAM1*, *VCAM1*, and *E-selectin* also showed no significant difference (adjust *P* < 0.05) in the TNFα-treated HUVEC with or without MLKL inhibition (Supplementary Fig. S10B–D), suggesting the downregulation of adhesion molecules expression was caused by other mechanisms than alternative splicing. Indeed, MLKL or RBM6 knockdown led to accelerated *ICAM1, VCAM1*, and *E-selectin* mRNA degradation in actinomycin-D-treated HUVEC (Fig. 6a, b). By contrast, RBM6 overexpression delayed the *ICAM1, VCAM1*, and *E-selectin* mRNA degradation (Fig. 6c). Thus, these data indicate that MLKL interacts with RBM6 and promotes *ICAM1*, *VCAM1*, and *E-selectin* expression by regulating adhesion molecule mRNA stability.

## Discussion

In this study, we identified a previously unknown function of MLKL in regulating endothelial adhesion molecules expression during TNFα-induced acute inflammation. MLKL, in cooperation with RBM6, potentiates ICAM1, VCAM1, and E-selectin expression by increasing mRNA stability in a necroptosis-independent manner. MLKL deficiency decreases the expression of adhesion molecules, reducing leukocyte adhesion and extravasation.

The prevailing dogma that MLKL is activated by RIPK3-mediated phosphorylation at T357/S358 to mediate necroptosis^3^, though additional modifications, such as phosphorylation at Y376 by TAM kinase^5^, may be still required for full activation of MLKL. We found that TNFα or TSZ treatment were not able to induce MLKL T357/S358 phosphorylation and trigger necroptosis in HUVEC due to extremely low expression level of RIPK3 (Fig. 1d, e). Only upon ectopic RIPK3 expression, TSZ treatment was able to induce necroptosis in HUVEC. Unexpectedly, we found that MLKL controlled adhesion molecule expression in HUVEC (Fig. 1e). Non-necroptotic function of MLKL has been recently observed in different conditions. In vitro studies and genetic evidence have demonstrated that MLKL controls NLRP3 inflammasome formation^10–12,33^. MLKL in injured nerve is phosphorylated at S441 by unknown kinases to degrade the myelin sheath^9^. Although, the mechanisms by which MLKL senses upstream signals in HUVEC, injured nerves are still not known, these studies and our data highlight that alternative mechanisms exist in controlling MLKL function, probably in a stimulus-specific and cell type-specific manner. In particular, kinases or proteins that activate MLKL and different forms of MLKL modifications are not fully understood and need to be characterized in the future.

We found that the majority of MLKL resided in the cytoplasm; with a small fraction located in the nucleus of unstimulated HUVEC and TNFα stimulation induced the accumulation of MLKL in the nucleus (Fig. 4a). Remarkably, the ectopic expression of nuclear-localized MLKL potently increased the adhesion molecule expression (Fig. 4c, d). Thus, our study supports the hypothesis that MLKL might regulate gene expression in the nucleus. However, MLKL does not contain any DNA or RNA binding domain, suggesting that it could not directly bind DNA or RNA and regulate gene expression. We found that MLKL interacted with an RNA-binding protein RBM6. It has been reported that RBM6 is involved in regulating RNA alternative splicing^31^. However, genome-wide or *ICAM1*, *VCAM1*, and *E-selectin* specific alternative splicing analysis revealed no apparent changes of alternative splicing upon MLKL inhibition (Supplementary Fig. S9). Indeed, we found that MLKL-RBM6 complex promoted adhesion molecule expression by increasing mRNA stability (Fig. 6a, b). However, the detailed mechanisms by which MLKL-RBM6 stabilizes or delays the degradation of adhesion molecule mRNA requires further investigation.

Endothelial cells form an important barrier that maintains tissue homeostasis by controlling liquid exchange and immune cell trafficking. Activation of endothelial cells results in the upregulation of adhesion molecules expression or disruption of vascular integrity, which contributes to the development of a variety of diseases, including inflammation and tumor metastasis^34,35^. Our data support previous studies that RIPK1 scaffolding function is required for the activation of the NF-*κ*B pathway (Fig. 3d), which controls the transcription of endothelial adhesion molecules. However, we found that MLKL did not affect NF-*κ*B activation. MLKL knockout mice are healthy and viable and show no vascular defects (Supplementary Fig. S4), suggesting that MLKL is not necessary for physiological vascular development^36,37^. Instead, MLKL regulated adhesion molecule expression at the post-transcriptional level by increasing mRNA stability upon TNFα stimulation. The deletion of MLKL led to significantly reduced leukocyte adhesion and extravasation during TNFα-induced acute inflammation (Fig. 2f–i).

We showed RIPK3 was expressed at an extremely low level in HUVEC, and its expression was not upregulated upon acute TNFα stimulation, which conferred HUVEC resistance to necroptosis. Lack of RIPK3 expression has also been reported in many other cell types, including hepatocyte and cancer cells^22,38^. However, we would like to point out that the expression of RIPK3 in HUVEC and other endothelial cells can be different due to organ-specific vascular heterogeneity. In addition, RIPK3 can be induced or upregulated in vivo under certain conditions, e.g., upon tumor cell injection^39^. Furthermore, genetic evidence showed that RIPK3 deficiency leads to reduced endothelial cell permeability or necroptosis, thereby suppressing tumor metastasis^39,40^. Nonetheless, the unique character of the low expression level of RIPK3 in HUVEC allowed us to delineate the non-necroptotic function of MLKL in endothelial cells in a simplified system.

**Fig. 7:**
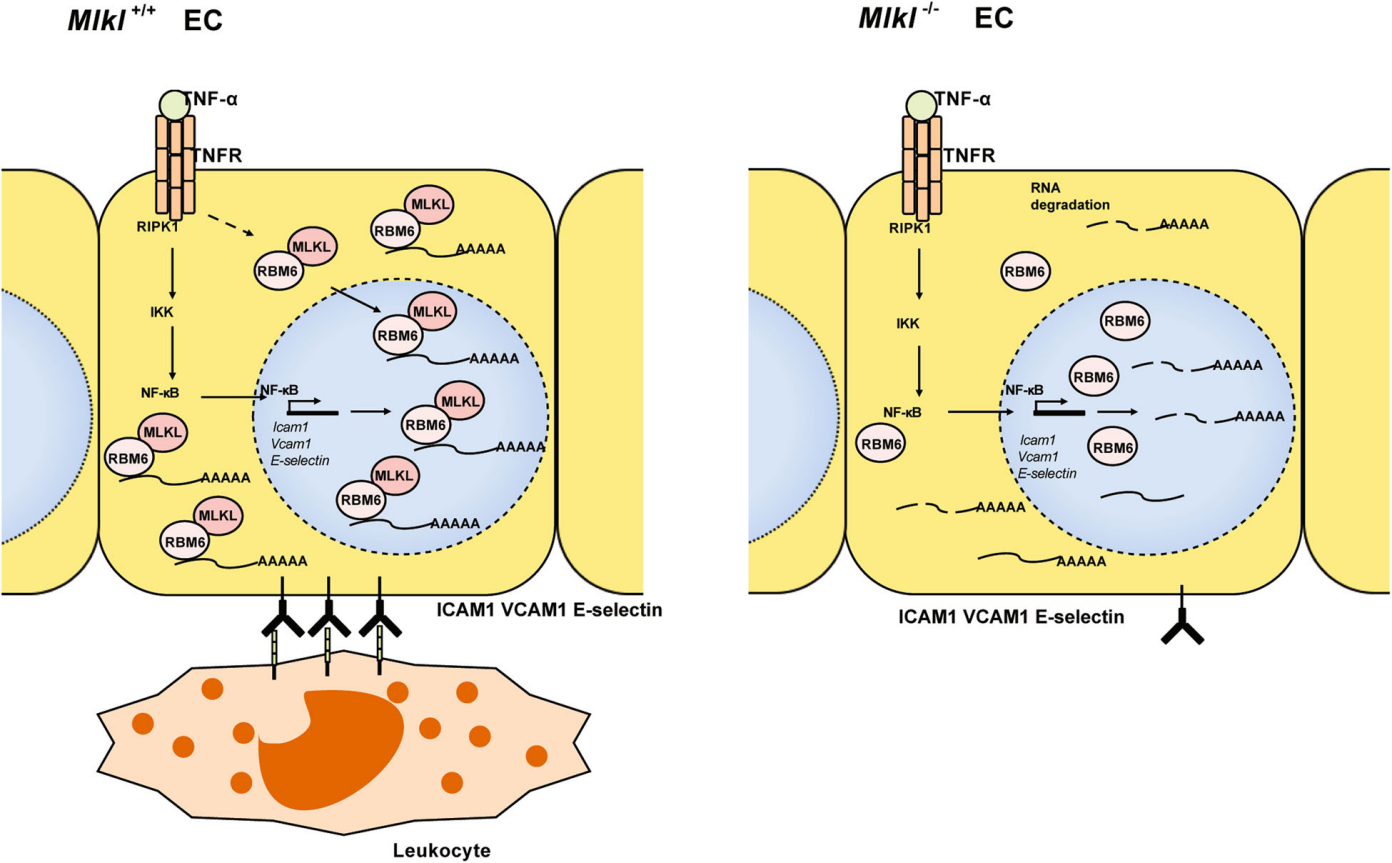
Working model: TNFα stimulation increases the association of MLKL and RBM6. Consequently, MLKL-RBM6 complex stabilizes the mRNA of ICAM1 VCAM1 and E-selectin and promotes the adhesion molecules protein expression.

In conclusion, the present study expands the current understanding of MLKL by demonstrating that MLKL, in association with RBM6, regulates endothelial adhesion molecule expression, consequently, the EC-leukocyte interaction independent of its necroptosis-executing function (Fig. 7). MLKL inhibition reduces immune cell infiltration into the injured tissue, thus, might synergize with its anti-necroptotic function and provides additional benefits for the treatment of inflammation-related diseases.

## Supplementary information

Supplementary figure legends

Fig. S1

Fig. S2

Fig. S3

Fig. S4

Fig. S5

Fig. S6

Fig. S7

Fig. S8

Fig. S9

Fig. S10

Table S1

Table S2

Movie S1

## Data Availability

The RNA-seq data were deposited in the Gene Expression Omnibus (GEO) database (Accession number: GSE142986).
