# Supplementary material for: A necroptotic-independent function of MLKL in regulating endothelial cell adhesion molecule expression
Source: Cell Death Dis. 2020 Apr 24;11(4):282. doi: 10.1038/s41419-020-2483-3 (PMC7181788; doi:10.1038/s41419-020-2483-3)
Supplement: Supplementary file 1 — Supplementary figure legends [file 41419_2020_2483_MOESM1_ESM.docx]

**Supplementary figure legends:**

**Fig. S1. MLKL and RIPK1, but not RIPK3, are highly expressed in HUVEC.**

(A) Real-time PCR validation of the mRNA levels of RIPK1, RIPK3, and MLKL in HUVECs. (B) HUVECs were treated with TNFα (100 ng/ml) for different periods of time. Protein levels of MLKL, RIPK1 and RIPK3 were determined by immunoblot. HT-29 serves as a positive control. (C) Immunoblot validation of the expression of RIPK3, RIPK1, and MLKL in HT-29, HUVEC, HDMVEC, iHDMVEC and HUAEC. (D) HUVECs and HT-29 were treated with TSZ for the indicated times. Necroptotic cells were visualized by PI staining. Hoechst 33342, blue; PI, red. Scale bar, 50 μm. Data are representative of three independent experiments.

**Fig. S2. MLKL inhibition reduces adhesion molecules expression in HUVEC.**

(A) Principal components analysis of gene expression of HUVECs treated with Vehicle, TNFα, NSA and TNFα+NSA (n=3 biological replicates). (B) The expression of ICAM1, VCAM1, E-selectin mRNA levels in HUVECs upon TNFα (50 ng/ml) treatment were analyzed by real-time PCR. (C) HUVECs were pretreated with NSA (4 μM) for 1 hr and then were further stimulated with TNFα (100 ng/ml) for 6 hr. Expression levels of adhesion molecules were determined by real-time PCR. Data (B, C) are representative of three independent experiments, with each experiment containing biological triplicates. Means ± SD; ***P < 0.001; Student’s t-test.

**Fig. S3. MLKL regulates adhesion molecules expression in MEF.**

(A) Immunoblot validation of knockout efficiency of MLKL in MEF. (B) Real-time PCR analysis of the mRNA levels of Icam1 in Mlkl+/+ and Mlkl-/- MEF upon TNFα (100 ng/ml) treatment. (C) Immunoblot validation of the reconstitution of mMLKL and hMLKL in Mlkl-/- MEF. (D) Real-time PCR analysis of the mRNA levels of Icam1 in Vector and mMlkl/hMLKL-reconstituted Mlkl-/- MEF treated with TNFα (100 ng/ml). Data are representative of three independent experiments, with each experiment containing biological triplicates. Means ± SD; **P < 0.01, ***P < 0.001; Student’s t-test.

**Fig. S4. MLKL deficiency does not affect vessel development.**

Retinas were isolated from Mlkl+/+ and Mlkl-/- mice pups at postnatal day 6 and stained with endothelial cell specific CD31 and pericyte specific Desmin antibodies. A: artery, V: vein.CD31, green; Desmin, red. Scale bar, 1 mm.

**Fig. S5. MLKL deficiency in leukocytes does not affect EC-leukocyte interaction.**

(A) Schematic depiction of local dermal inflammation model, in which TNFα (100 ng in 50 μL) or equal volume of saline were intradermally injected into right and left skin flap respectively. (B-C) The percentage of CD18, CD11b, CD49d, CD162 expressing leukocytes amongst CD45+ leukocytes isolated from peripheral blood of Mlkl+/+ and Mlkl-/- mice were analyzed with flow cytometry. Representative FACS plots were shown in (B) and quantification were shown in (C) (Mlkl+/+=3, Mlkl-/-=4). (D) The mRNA expression levels of Cd18, Cd11b, Cd49d, and Cd162 in the CD45+ leukocytes of Mlkl+/+ and Mlkl-/- mice were determined by real-time PCR. (E, F) Calcein-AM labelled CD45+ leukocytes freshly isolated from peripheral blood of Mlkl+/+ and Mlkl-/- were incubated with TNFα-primed HUVEC monolayers. Adherent leukocytes were imaged (E) and quantified (F). Data (E, F) are representative of three independent experiments, with each experiment containing biological triplicates. Means ± SD; ns, not significant; Student’s t-test. Scale bar, 100 μm.

**Fig. S6. RIPK1 scaffolding function, not its kinase activity, regulates Icam1 expression.**

(A) MEFs were pretreated with Nec-1s (10 μM) for 1 hr and then followed by TNFα (100 ng/ml) treatment for 6 hr. Expression level of Icam1 was determined by real-time PCR. (B) Immunoblot validation of RIPK1 deficiency in the MEFs. (C) Real-time PCR analysis of the mRNA level of Icam1 in the Ripk1+/+ and Ripk1-/- MEFs stimulated with TNFα. Data are representative of three independent experiments, with each experiment containing biological triplicates.Means ± SD; ***P < 0.001; ns, not significant; Student’s t-test.

**Fig. S7. MLKL does not crosstalk with NF-κB pathway.**

(A-C) HUVECs were stimulated with TNFα (50 ng/ml) for the indicated times. The cell lysates were immunoprecipitated with anti-TNFR (A), anti-NEMO (B) and anti-MLKL (C) antibodies, and the immunocomplexes were immunobloted with the indicated antibodies. (D) Control and MLKL knockout HUVECs were stimulated with TNFα (50 ng/ml) for 15 min. p65 nuclear translocation was determined by immunofluorescent staining. Hoechst 33342, blue; Phalloidin, red; p65, green. Scale bar, 20 μm. Data are representative of three independent experiments.

**Fig. S8. MLKL does not interact with KINH, PGAM5 and S10A8.**

(A-C) HUVECs that were lentivirally transduced with shRNAs targeting KINH (A), PGAM5 (B) and S10A8 (C) were stimulated with TNFα (50 ng/ml) for 6 hr. Knockdown efficiencies of KINH, PGAM5 and S10A8 were confirmed by real-time PCR. ICAM1 and VCAM1 expression levels upon KINH, PGAM5 and S10A8 knockdown were analyzed by immunoblotting. Data are representative of three independent experiments.

**Fig. S9 Intracellular localization of MLKL and RBM6.**

HUVEC were lentivirally transduced with HA-MLKL and Flag-RBM6 followed by TNFα (50 ng/ml) treatment for 6 h. Intracellular distribution of HA-MLKL and Flag-RBM6 was determined by immunofluorescence staining (A). Hoechst, blue; Flag, green; HA, red. Scale bar, 20 μm.

**Fig. S10. MLKL inhibition does not affect alternaltive RNA splicing.**

(A) The number and percentage of different types of alternative splicing events in TNFα and NSA+TNFα-treated HUVECs. (Β-D) Alternative exon usage of ICAM1, VCAM1 and E-selectin in TNFα and NSA+TNFα-treated HUVECs. The upper panel shows the normalized expression level of each exon of ICAM1 in TNFα and NSA+TNFα-treated HUVECs; Lower panel shows transcript isoform annotation of ICAM1 from Ensembl.

**Table S1.** The list of differentially expressed genes shown in the heatmap of **Figure 2A**.

**Table S2.** Sequence of the primers used in this study.

**Movie S1.** Intravital imaging of leukocyte adhesion in the skin of MLKL^+/+^ and MLKL^-/-^ mice after TNFα stimulation.
