# Supplementary figures and images for: A necroptotic-independent function of MLKL in regulating endothelial cell adhesion molecule expression
Source: Cell Death Dis. 2020 Apr 24;11(4):282. doi: 10.1038/s41419-020-2483-3 (PMC7181788; doi:10.1038/s41419-020-2483-3)

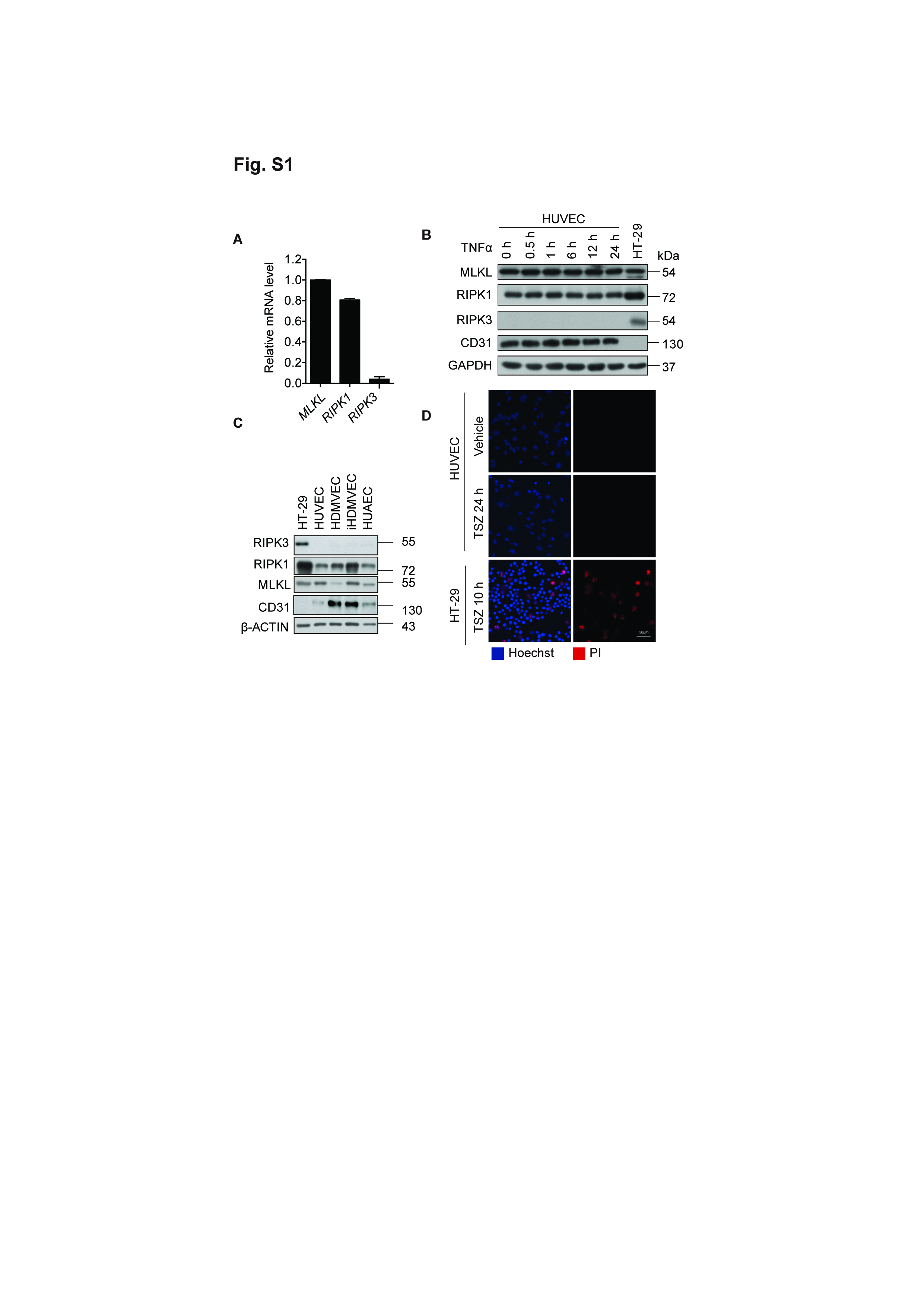

Supplement: Supplementary file 2 — Fig. S1 [file 41419_2020_2483_MOESM2_ESM.tif]

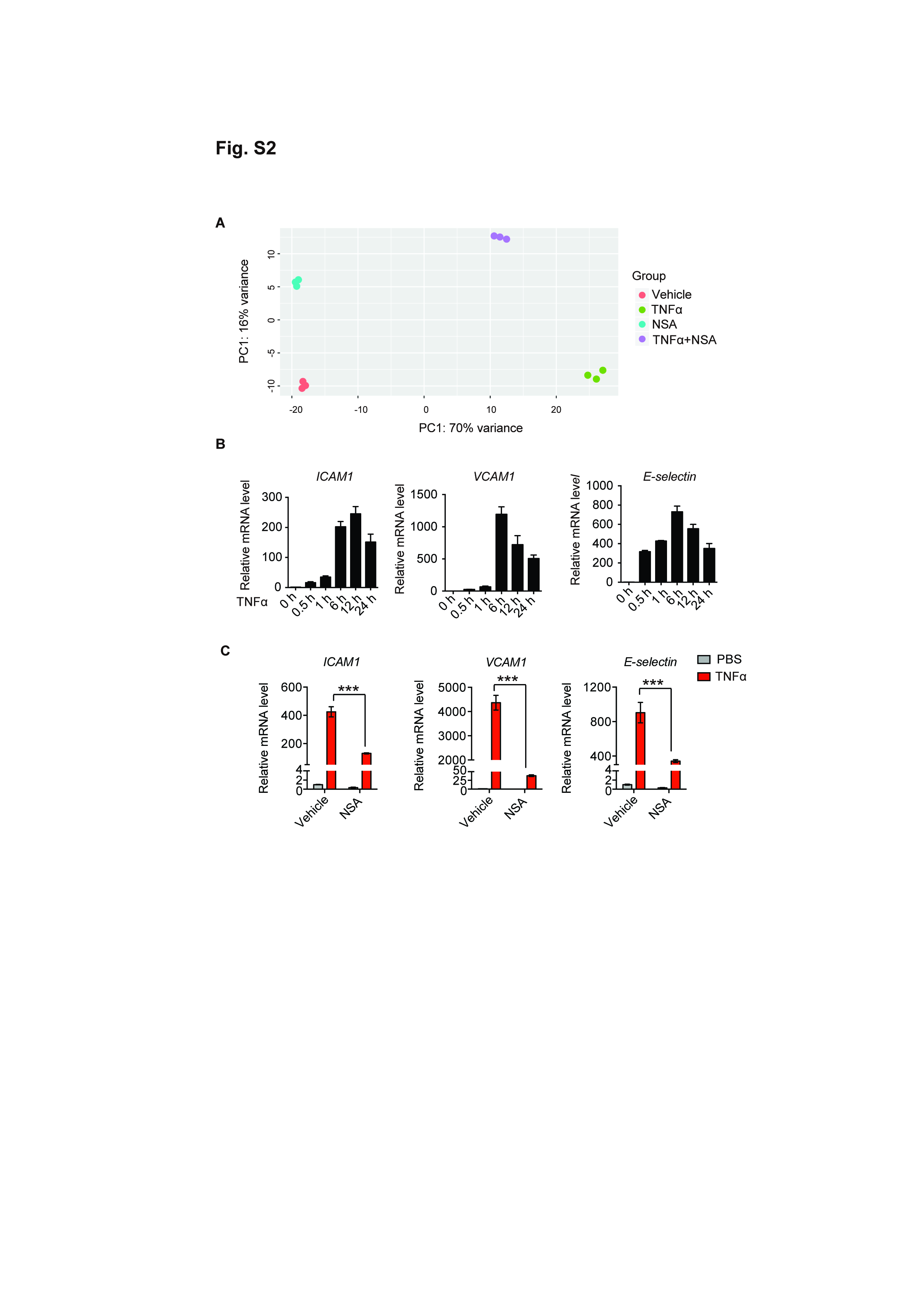

Supplement: Supplementary file 3 — Fig. S2 [file 41419_2020_2483_MOESM3_ESM.tif]

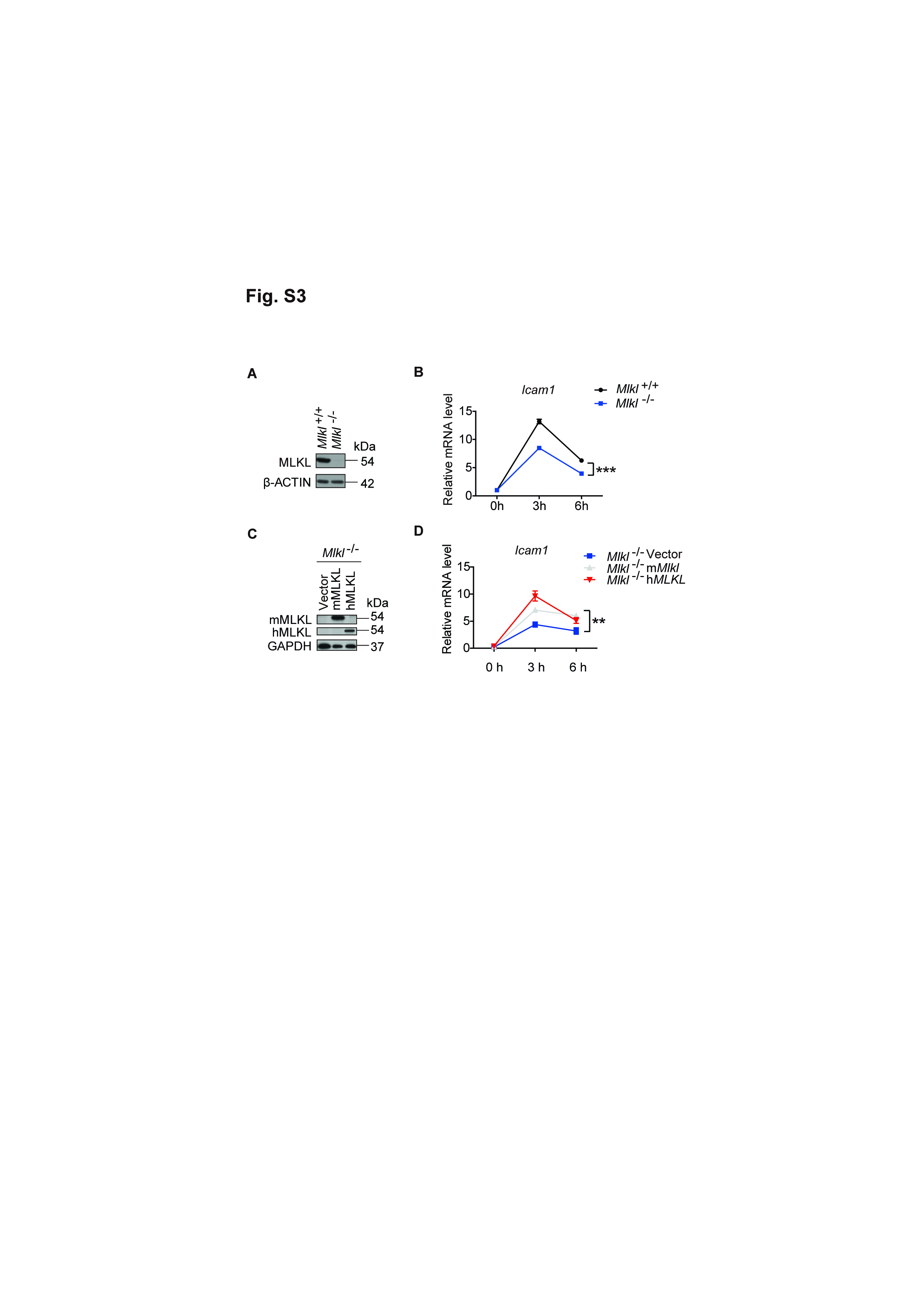

Supplement: Supplementary file 4 — Fig. S3 [file 41419_2020_2483_MOESM4_ESM.tif]

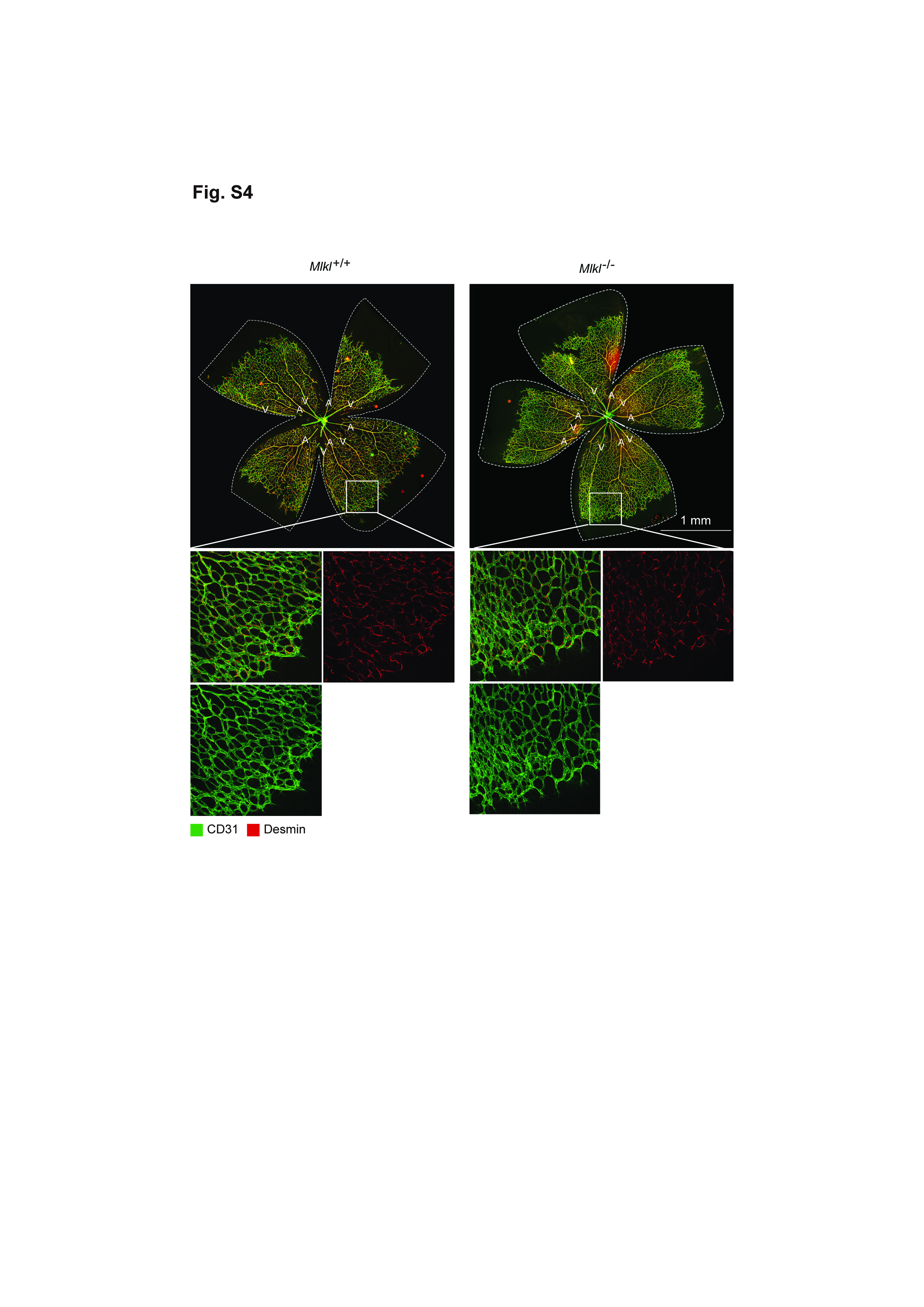

Supplement: Supplementary file 5 — Fig. S4 [file 41419_2020_2483_MOESM5_ESM.tif]

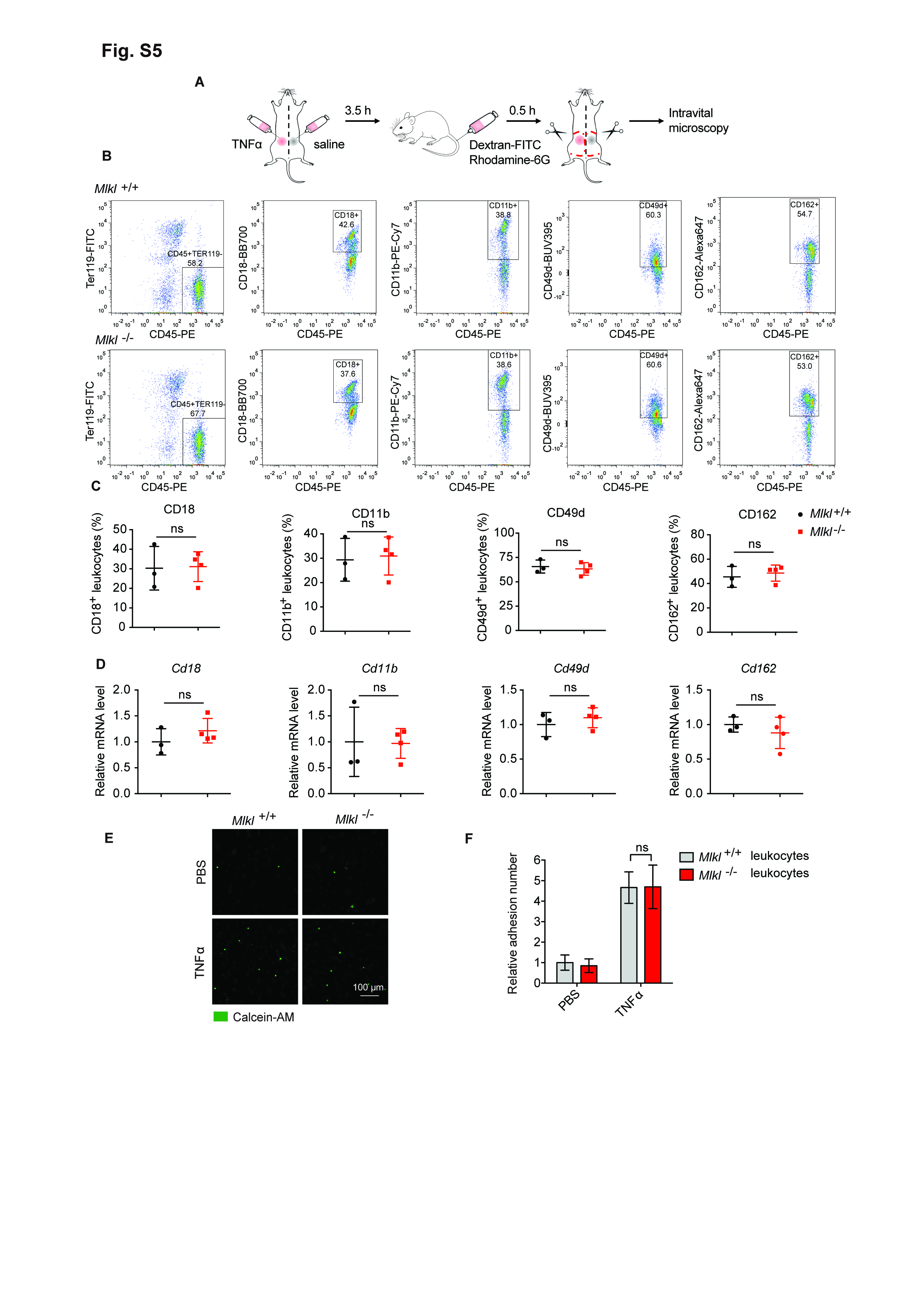

Supplement: Supplementary file 6 — Fig. S5 [file 41419_2020_2483_MOESM6_ESM.tif]

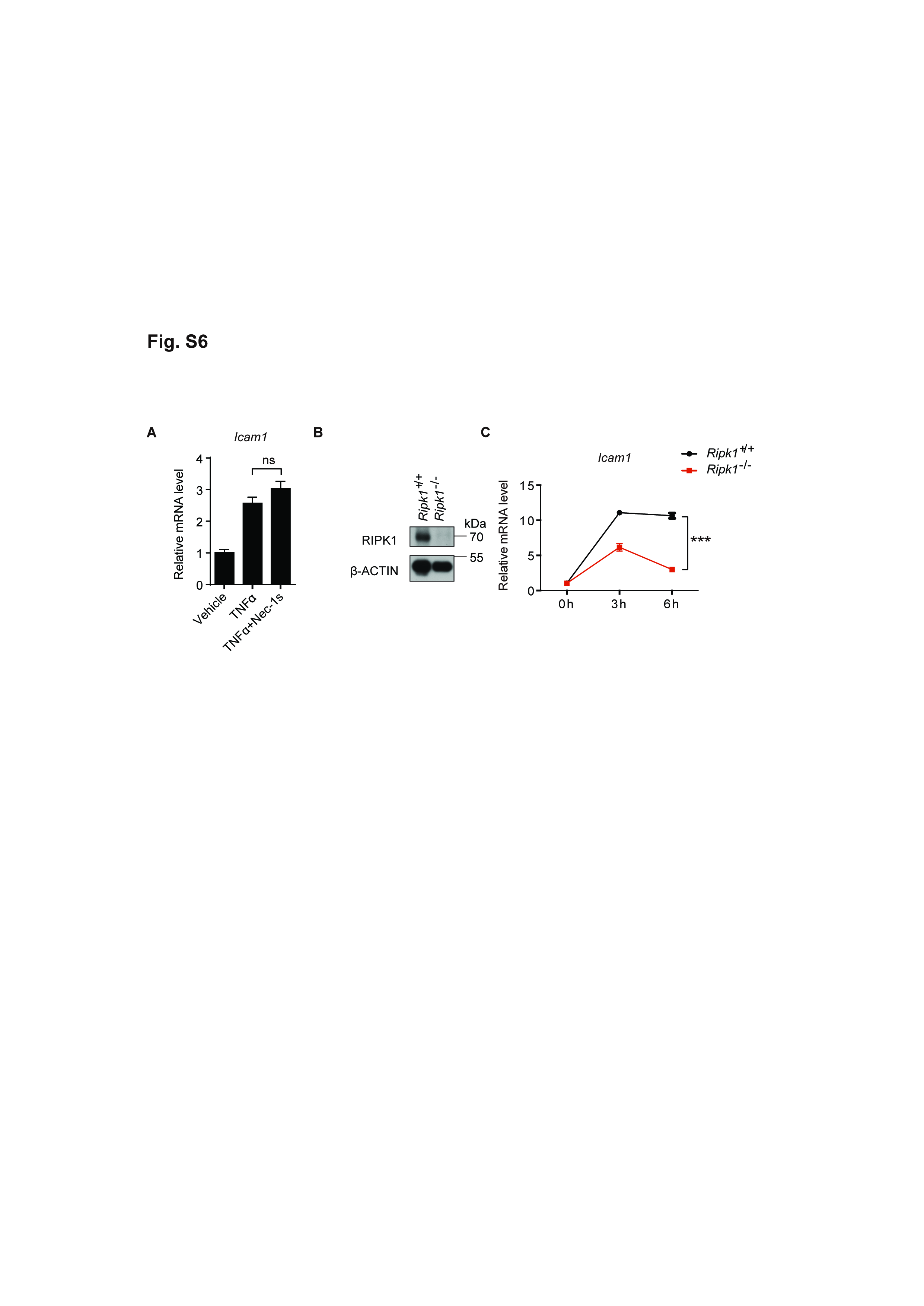

Supplement: Supplementary file 7 — Fig. S6 [file 41419_2020_2483_MOESM7_ESM.tif]

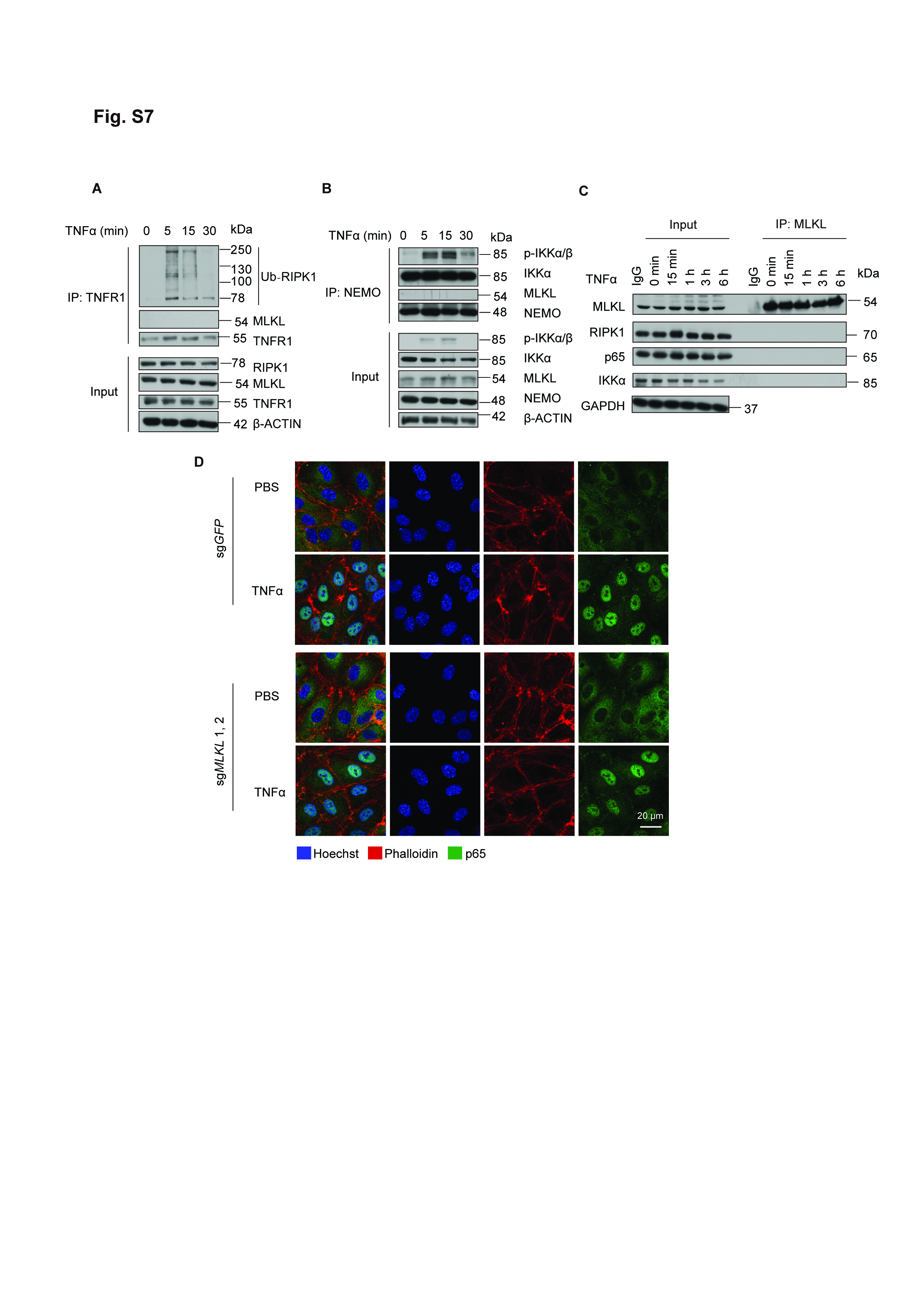

Supplement: Supplementary file 8 — Fig. S7 [file 41419_2020_2483_MOESM8_ESM.tif]

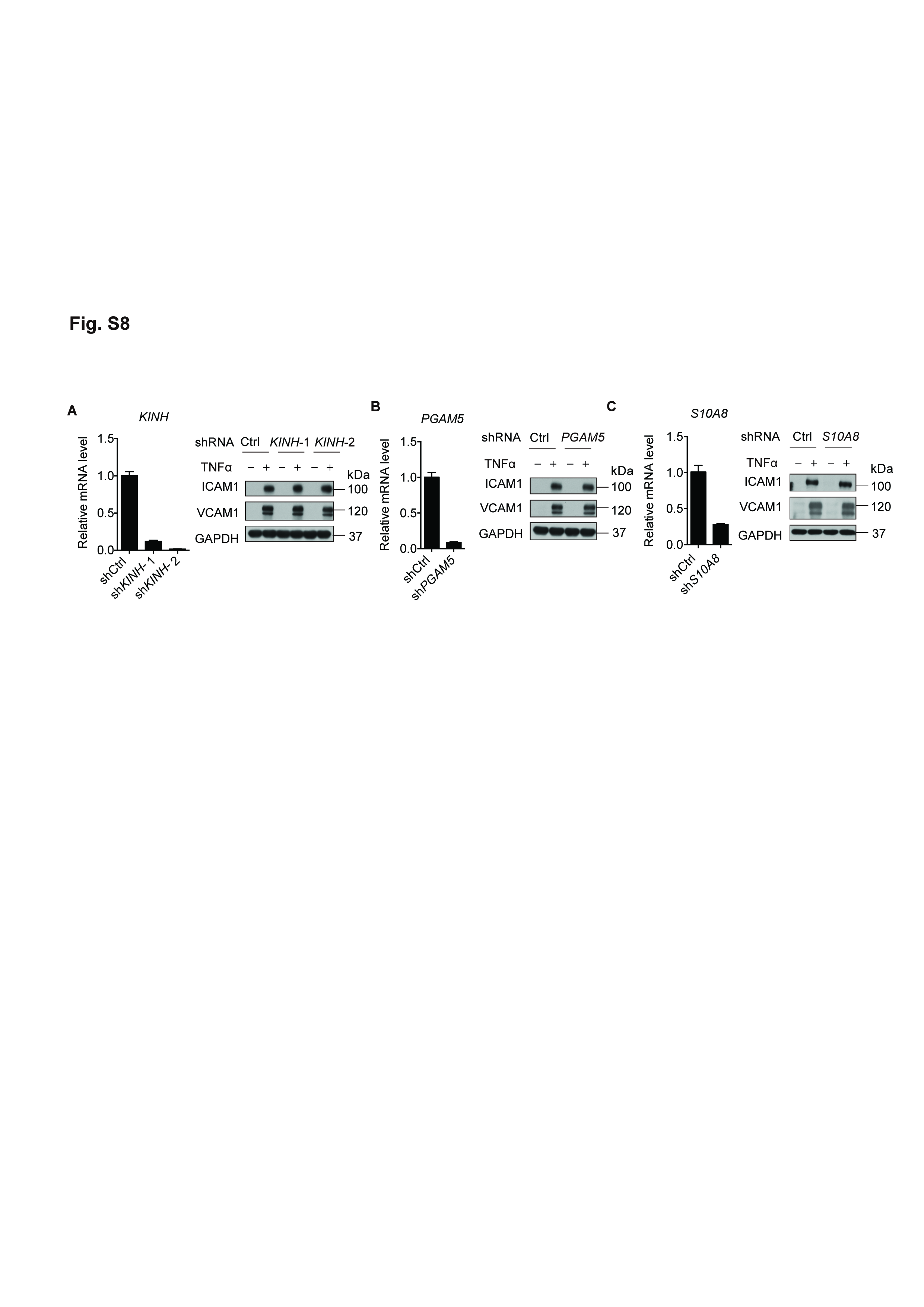

Supplement: Supplementary file 9 — Fig. S8 [file 41419_2020_2483_MOESM9_ESM.tif]

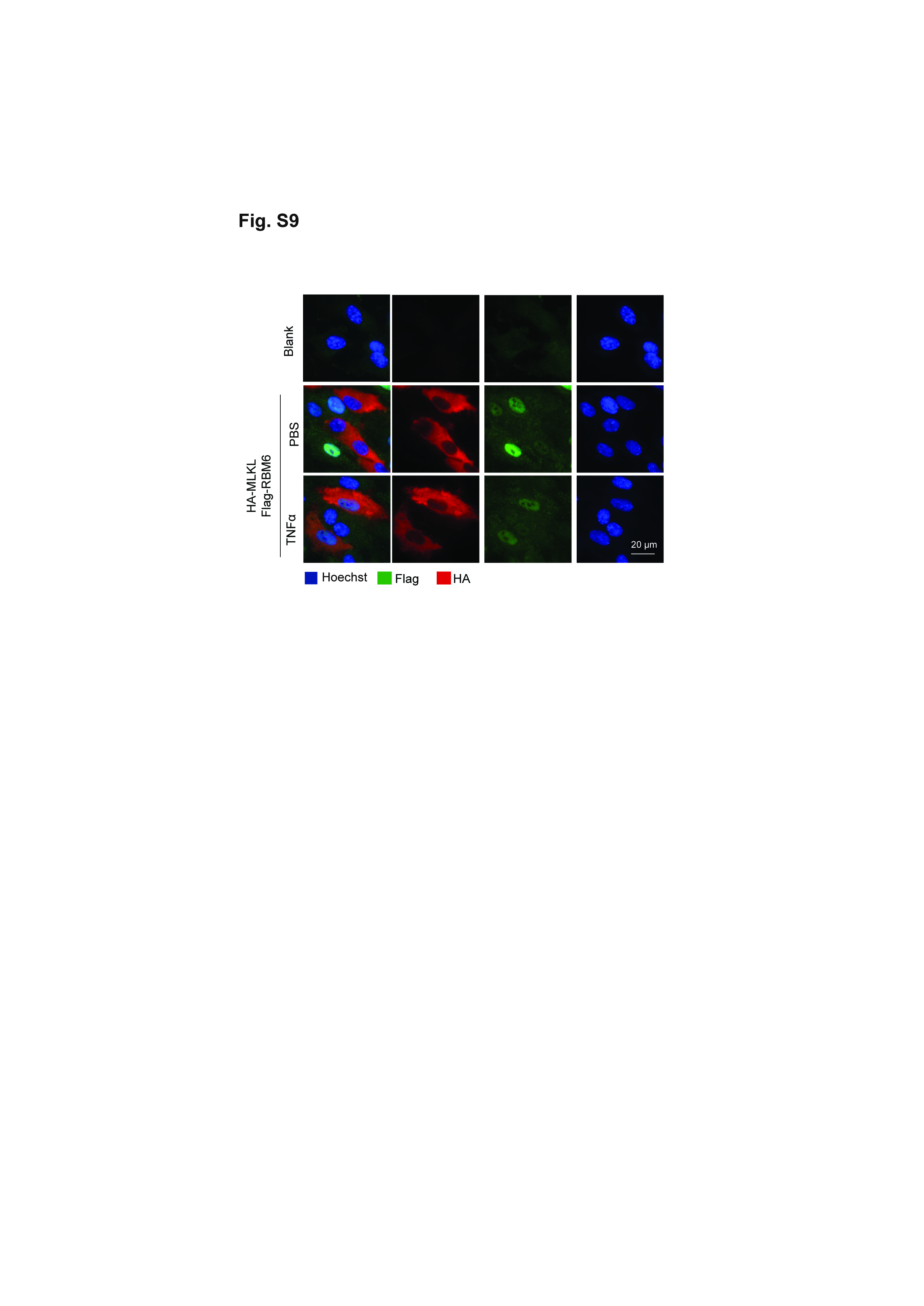

Supplement: Supplementary file 10 — Fig. S9 [file 41419_2020_2483_MOESM10_ESM.tif]

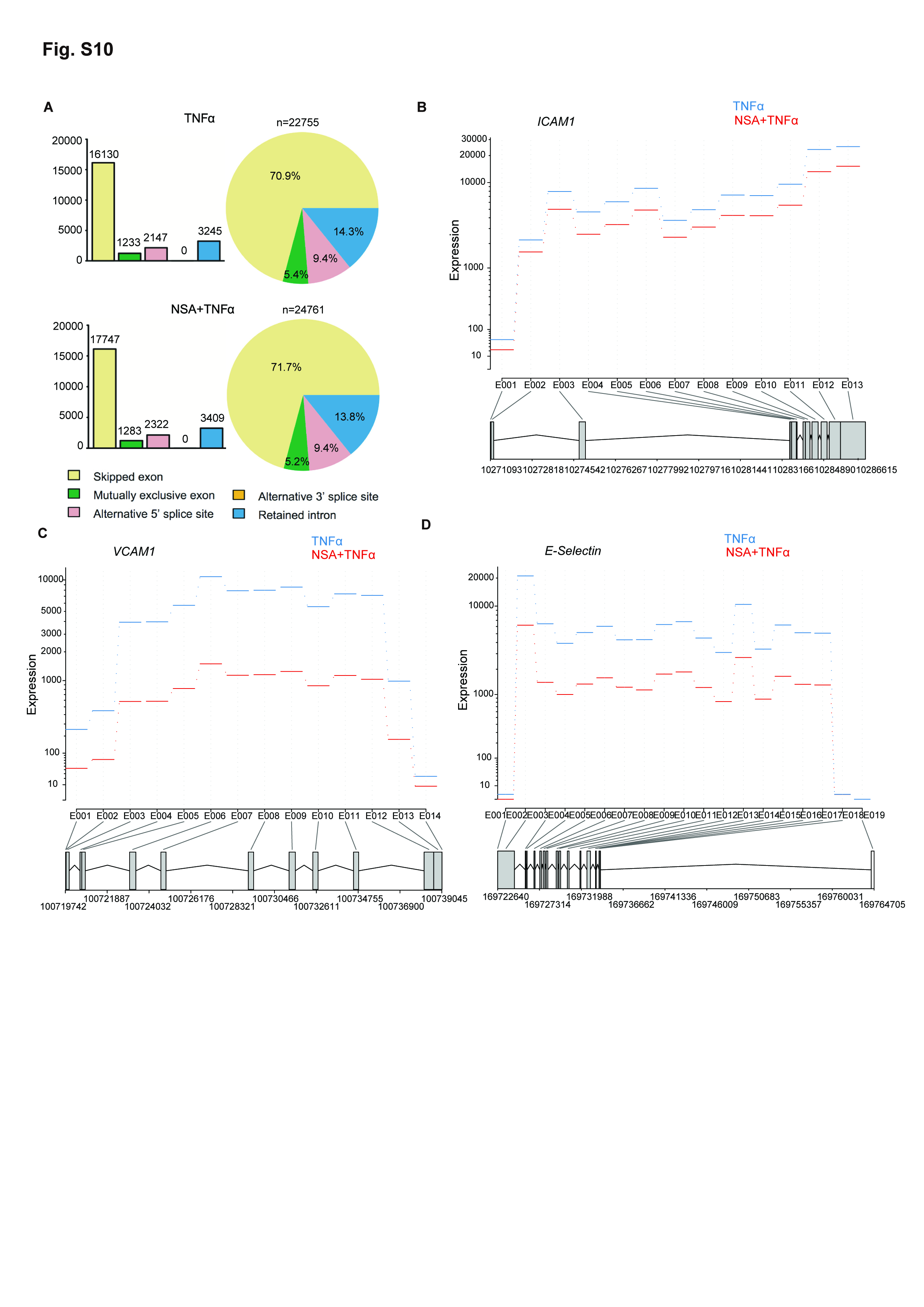

Supplement: Supplementary file 11 — Fig. S10 [file 41419_2020_2483_MOESM11_ESM.tif]
